# Integrated multiomics analysis of chromosome 19 miRNA cluster in bladder cancer

**DOI:** 10.1007/s10142-023-01191-0

**Published:** 2023-08-05

**Authors:** Akshay Pramod Ware, Kapaettu Satyamoorthy, Bobby Paul

**Affiliations:** 1 Department of Bioinformatics, Manipal School of Life Sciences, Manipal Academy of Higher Education, Manipal, Karnataka 576104 India; 2 Department of Cell and Molecular Biology, Manipal School of Life Sciences, Manipal Academy of Higher Education, Manipal, Karnataka 576104 India; 3 SDM College of Medical Sciences and Hospital, Shri Dharmasthala Manjunatheshwara (SDM) University, Manjushree Nagar, Sattur, Dharwad, Karnataka 580009 India

**Keywords:** Cancer biomarker, Epigenetics, Carcinogenesis, Cancer informatics

## Abstract

With 46 microRNAs (miRNAs) embedded tandemly over a distance of ~100 kb, chromosome 19 microRNA cluster (C19MC) is the largest miRNA cluster in the human genome. The C19MC is transcribed from a long noncoding genomic region and is usually expressed simultaneously at a higher level. Hence, we performed an integrative multiomics data analysis to examine C19MC regulation, expression patterns, and their impact on bladder cancer (BCa). We found that 43 members of C19MC were highly expressed in BCa. However, its co-localization with recurrent copy number variation (CNV) gain was not statistically significant to implicate its upregulation. It has been reported that C19MC expression is regulated by a well-established CpG island situated 17.6 kb upstream of the transcription start site, but we found that CpG probes at this island were hypomethylated, which was not statistically significant in the BCa cohort. In addition, the promoter region of C19MC is strongly regulated by a group of seven transcription factors (*NR2F6*, *SREBF1*, *TBP*, *GATA3*, *GABPB1*, *ETV4*, and *ZNF444*) and five chromatin modifiers (*SMC3*, *KDMA1*, *EZH2*, *RAD21*, and *CHD7*). Interestingly, these 12 genes were found to be overexpressed in BCa patients. Further, C19MC targeted 42 tumor suppressor (TS) genes that were downregulated, of which 15 were significantly correlated with patient survival. Our findings suggest that transcription factors and chromatin modifiers at the promoter region may regulate C19MC overexpression. The upregulated C19MC members, transcription regulators, and TS genes can be further exploited as potential diagnostic and prognostic indicators as well as for therapeutic management of BCa.

**Supplementary Information:**

The online version contains supplementary material available at 10.1007/s10142-023-01191-0.

## Introduction

Bladder cancer (BCa) is the most prevalent urothelial neoplasm, with an anticipated 573,278 cases and 212,548 deaths globally in 2020 (Sung et al. 2021). BCa is routinely diagnosed using imaging, cystoscopy, and urine cytology tests. However, these fall short of expectations due to poor sensitivity, high cost, and invasive procedures (Su et al. 2019; Yang and Zhang 2023). The current diagnostic approaches are also unsuitable for early BCa detection due to lack of sufficient sensitivity and specificity for diagnosis, especially in low-grade cancer cases (Charpentier et al. 2021). Transurethral surgery and intravesical chemotherapy with or without radiation are often used to treat BCa patients (Tran et al. 2021). There have been several advances in the monitoring and treatment of BCa and patients are expected to show prolonged survival (Tran et al. 2021). Therefore, identifying novel biomarkers is essential to improve diagnostic, prognostic, and therapeutic approaches (Chowdhury et al. 2023; Loganathan and Doss 2023).

The miRNAs are often present in the genome as clusters. Chromosome 19 microRNA cluster (C19MC) is the largest in the human genome, consists of 46 miRNAs spanning ~100 kb, and is located at the cytoband chr19q13.42 (Bentwich et al. 2005). The C19MC is a primate-specific imprinted miRNA cluster that emerged late in the evolution of the primate lineage, and a bioinformatics study suggested that it plays essential roles in primate reproduction, development, and differentiation (Lin et al. 2010). Cancers of the breast (Jinesh et al. 2018), brain (Sin-Chan et al. 2019), thyroid (Rippe et al. 2010), and infantile hemangioma (Strub et al. 2016) have already been linked to the overexpression of C19MC members. There are few studies on the function of individual members of the C19MC in BCa. For example, mir-516a can promote BCa metastasis by targeting *MMP9 via* the AKT/FOXO3A/SMURF1 axis (Chang et al. 2020). Another study reported that restoring mir-517a expression causes cell apoptosis in bladder cancer cell lines (Yoshitomi et al. 2011).

C19MC members are classified into three subgroups based on sequence similarity to better comprehend the reasons for selective activation of C19MC in various cancer types (Nguyen et al. 2017). The sequence alignment of C19MC miRNAs revealed that 16 of them share the same hexameric seed sequence (5′-AAGUGC-3′) with members of the mir-302/-372 miRNA cluster, which is known to positively affect pluripotency induction and stemness maintenance in mesenchymal stem cells (Subramanyam et al. 2011). The C19MC-AAGUGC-miRNA group was shown to contribute more effectively in gene targeting and silencing (Nguyen et al. 2017). As a result of the significant sequence similarity in the seed regions, miRNA clusters generally target the same or related genes within the same pathway. Hence, it is more important to investigate the impact of aberrant expression of clustered miRNAs than that of nonclustered miRNAs.

Changes in DNA copy number, mutations, epigenetic modifications, dysregulation of transcription factors (TFs), and alterations in the miRNA biogenesis pathway can cause aberrant miRNA expression during carcinogenesis (Syeda et al. 2020). The co-localization of copy number variations (CNVs) and miRNAs has demonstrated the potential mechanism behind C19MC miRNA dosage (Vaira et al. 2012). Lambo et al. (2020) have shown that C19MC has oncogenic effects in embryonal tumor with multilayered rosettes (ETMRs). Furthermore, gene fusion involving the *TTYH1* promoter and C19MC leads to abnormally high C19MC expression, which has been observed in brain-specific malignant tumors (Kleinman et al. 2014). The C19MC cluster transcription is highly correlated with the CpG island 17.6 kb upstream in the promoter region in various cancer cell lines (Tsai et al. 2009). In contrast, a study by Jinesh et al. (2018) showed that high C19MC expression was not significantly correlated with hypomethylation of CpG islands and suggested regulation of C19MC expression in human breast cancer is not primarily determined by methylation at CpG-islands.

However, the biological role of C19MC members in BCa is largely unknown. Hence, the study integrated publicly available multiomics datasets to understand the regulation, expression pattern, and biological role of C19MC candidates in BCa progression. We investigated the role of recurrent copy number variations (RCNVs), promoter DNA methylation, and histone modifications (HMs) in the regulation of C19MC members. We also examined the transcriptional regulators that are involved in the control of C19MC expression. The comprehensive multiomics data analysis provides a better understanding of C19MC regulation in BCa and new perspectives on C19MC as a diagnostic and therapeutic target.

## Materials and methods

### Data acquisition

The detailed information on C19MC was retrieved from the miRBase v22.1 (Kozomara et al. 2019). The miRNASeq, RNASeq, and clinical data (level 3) were retrieved from the TCGA-BLCA cohort (https://portal.gdc.cancer.gov/) with the help of the R package TCGAbiolinks (Colaprico et al. 2016). Subsequently, C19MC member expression datasets from 19 cancer types (BLCA, BRCA, CESC, CHOL, COAD, ESCA, HNSC, KICH, KIRC, KIRP, LIHC, LUAD, LUSC, PCPG, PRAD, READ, STAD, THCA, and UCEC) along with normal samples were retrieved to conduct a pan-cancer analysis. The details of chromosome fragile sites (CFSs) on the Chr19 and their genomic coordinates were obtained from the HumCFS database (Kumar et al. 2019). The study used the hg38 genome build, and the UCSC LiftOver (https://genome.ucsc.edu/cgi-bin/hgLiftOver) tool was used to convert genome coordinates between assemblies wherever needed.

### Integrated C19MC, CNV, CFS, and 450k methylation array analysis

We executed CmiRClustFinder v2.0 (Ware et al. 2022a) pipeline to identify recurrent copy number variations (RCNVs) from BCa patient data. The genome coordinates of C19MC in BED file format were given as input to identify their co-localization with significant RCNV regions. The BCa 450k methylation array data for CpG probes targeting pre-C19MC region (Chr19:53596679-53666679; 70kb) were extracted from the Wanderer web server (http://maplab.imppc.org/wanderer/), which provides DNA methylation data from TCGA studies (Díez-Villanueva et al. 2015).

### C19MC expression pattern analysis in BCa patients

To further comprehend the C19MC miRNA expression profile among BCa patients, we conducted a correlation analysis using TCGA miRNASeq data. Additionally, we followed the standard procedure of the TCGAbiolink package for miRNA and mRNA differential expression analysis (DEA). The *TCGAnalyze_Preprocessing* function was used for outlier checking and sample correlation. The *TCGAnalyze_Normalization* function was used for normalization using both GC content and gene length, and genes were also filtered with the *TCGAnalyze_Filtering* function. According to a previous study (Bullard et al. 2010), we applied the DEA only to mRNAs and miRNAs with higher than the 0.25 quantile means. The function “glmLRT” was used to calculate the pairwise differentially expressed miRNAs (DEmiRs) and genes (DEGs) between normal and tumor samples. DEmiRs and DEGs were accessed by the function *TCGAnalyze_DEA* considering Log2Fold change > 1.5 and FDR < 0.01 threshold.

### Identification of potential transcriptional regulators

To further explore the transcriptional regulators that may influence C19MC expression, we used the UCSC Genome Browser (Kent et al. 2002) to retrieve TF binding sites, H3K27Ac marks, and enhancer/promoter elements around pre-C19MC (Chr19:53656679-53666679), C19MC (Chr19:53666679-53762430), and post-C19MC (Chr19:53666679-53676679) regions. Based on the literature, the identified genes were classified as transcription activators, transcription repressors, and chromatin modifiers. Furthermore, to confirm the transcriptional activity of selected TFs, they were cross-checked in the AnimalTFDB4 database (Hu et al. 2019).

### Grouping and integration of C19MC and CNV datasets

The miRNASeq dataset of BCa patients was processed to obtain the cumulative expression of all 46 miRNAs belonging to the C19MC. Cumulative expression of C19MC in normal bladder samples (*n*=19) was employed to fix a cut-off for overexpression (>77.89 raw reads). The BCa samples were categorized into two groups, C19MC^High^ (>cut-off) and C19MC^Low^ (<cut-off), and each group contained 201 samples. The segment mean values from CNV datasets were used to categorize high (>0.2) and low (<−0.2) CNV patient groups. We compared the CNV^High^ with C19MC^High^ and CNV^Low^ with C19MC^Low^ groups to better understand the role of CNV in C19MC regulation.

### Mapping of genes targeted by C19MC members

The miRNA-gene network was constructed including experimentally validated and predicted miRNA-gene interactions. The R package linked with 14 databases “multiMiR” (Ru et al. 2014) was used to mine computationally predicted and experimentally validated C19MC target genes. The study included only the targets of C19MC listed in at least two miRNA-gene interaction databases. We identified C19MC-targeted and differentially expressed genes by integrating each C19MC target gene and expression information. Furthermore, the search was narrowed down to the top 10% (based on the multiMiR score) of all target genes. A total of 616 unique target genes were ranked based on the number of miRNAs that targeted them.

### Effect of upregulated C19MC expression on tumor suppressor (TS) genes

A catalogue of 493 TS genes reported in BCa was obtained from the TSGene (Zhao et al. 2013) database and then Venn analysis was performed with 616 C19MC targets. The differential expression (normal vs. tumor) box plots for TS genes were procured from the web server for gene expression profiling and interactive analysis (Tang et al. 2017). Furthermore, the patients were categorized as having low or high expression levels to identify prognostically relevant genes based on median gene expression levels. Overall survival (OS) was calculated using the Kaplan–Meier plotter (Nagy et al. 2021) with various clinical parameters.

### Network construction of C19MC and inversely correlated targets

The effect of C19MC’s high levels of expression on its target genes was investigated using inversely correlated (i.e., upregulated miRNA and downregulated targeted mRNA) miRNA-mRNA pairs. In addition, we examined the CNV co-localization and methylation status of all target genes to confirm that they were solely downregulated by C19MC activity. Target genes associated with RCNV and their methylation status were obtained by executing the CmiRClustFinder v1.0 pipeline and mining the DNMIVD (Ding et al. 2020) database, respectively. Genes that were either co-localized with CNV deleted regions or hypermethylated in BCa patients were eliminated from the analysis. To predict the functional protein association networks among the remaining genes, the C19MC targets were mapped to the STRING v11.5 (https://string-db.org/) with a medium confidence score ≥ 0.40. The Molecular Complex Detection (MCODE) plugin from the Cytoscape (Bader and Hogue 2003) was utilized to distinguish the module that best represents the clusters of target genes. Strict cut-off requirements were applied (degree cut-off = 2; node score cut-off = 0.2; k-core = 2; max depth = 100). The ToppGene module (Chen et al. 2009) was used to prioritize, generate, and visualize gene networks based on GO-enrichment analysis (GOEA). The BioLayout Express^3D^ (Theocharidis et al. 2009) tool was used for module visualization. Subsequently, the 15 most highly connected hub genes were determined using the CytoHubba (Chin et al. 2014) plugin of Cytoscape.

### Functional enrichment analysis

The online portal WebGestalt (Liao et al. 2019) was used to perform gene functional enrichment analysis of C19MC target genes in order to identify their molecular function (MF), biological process (BP) participation, and cellular component (CC) localization. Gene enrichment analysis was also carried out using the KEGG pathway. The top genes were identified with a false discovery rate (FDR) of < 0. 05 considered.

### Estimation of tumor-infiltrating immune cells

The percentages of infiltrating immune cells were calculated using the “Cell-type Identification by Estimating Relative Subsets of RNA Transcripts (CIBERSORT)” method (Newman et al. 2015). Based on standardized gene expression data, the CIBERSORT deconvolution method can compute the cell composition of complicated tissues. The gene expression matrix (*n*=616 genes) data were uploaded to the CIBERSORT online portal, and fractions of 22 different types of immune cells were calculated using the default signature matrix with 1000 permutations. The study visualized the matrix of 22 immune cell fractions from 182 tumor samples with significant *p* values.

### Statistical analysis

The R studio (version 4.1.2) was used for all statistical analyses. The Pearson correlation coefficient was used to assess the correlation between 22 immune cells. The correlation matrix was visualized with the Corrplot v0.92 package. To analyze the differences between the normal and tumor groups, an unpaired, nonparametric Mann-Whitney test was utilized. A *p* value < 0.05 was considered statistically significant throughout the study.

## Results

### Integrative analysis and overall statistics

**Fig. 1:**
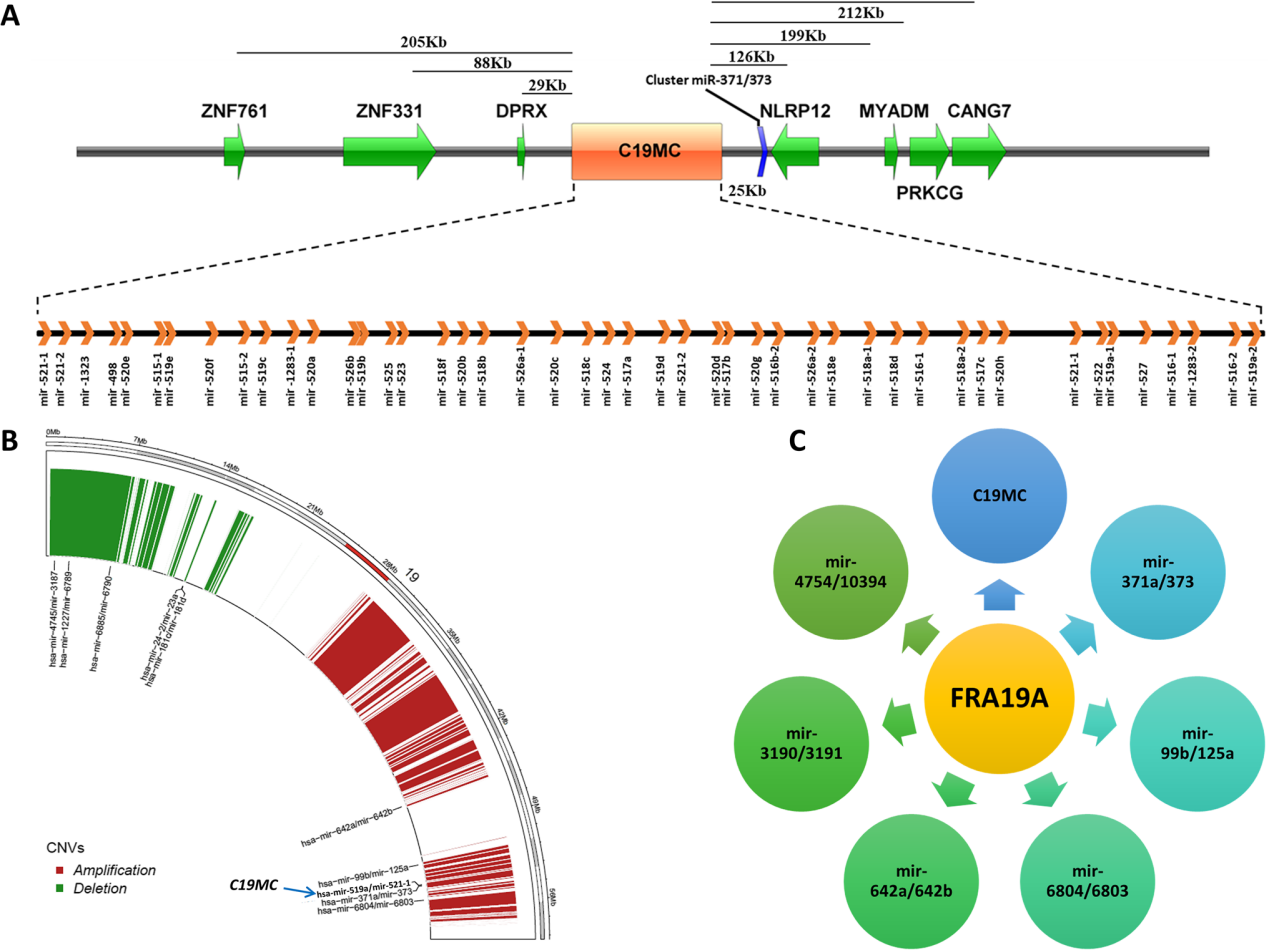
**A** Genomic organization of the C19MC miRNA cluster. Protein-coding genes are represented by green color, whereas the location of C19MC is given as orange. At the bottom, an enlargement shows the position of individual miRNA genes. Gene symbols refer to the following protein-coding genes: ZNF761—zinc finger protein 761, ZNF331—zinc finger protein 331, DPRX—divergent-paired related homeobox, NLRP12—NLR family, pyrin domain-containing 12, MYADM—myeloid-associated differentiation marker, PRKCG—protein kinase C gamma. **B** Circos plot of co-localization of C19MC cluster with recurrent CNV region (Chr19:53530432-54088052) in TCGA bladder cancer patient dataset. Other miRNA clusters associated with CNV regions are also shown. **C** C19MC is associated with the common chromosome fragile site FRA19A (Chr19:32400001-59128983) with the other six miRNA clusters

The analysis of miRNASeq and RNASeq datasets between normal and BCa samples resulted differentially expressed 694 miRNAs and 1808 mRNAs (Supplementary Table S1 & S2). We have identified 167 significant RCNV aberrations on chromosome 19 (Chr19) across the 408 BCa samples analyzed. Of these, 116 regions were amplified and 51 regions were deleted (Supplementary Table S3). The C19MC is located on Chr19, and it consists of 46 miRNA genes that span over ∼100 kb long region (Chr19:53666679-53762430). Figure 1A illustrates the genomic arrangement of C19MC with adjacent genes. The mapping of C19MC coordinates on RCNV regions revealed that C19MC is co-localized with the CNV gain region in BCa patients (Fig. 1B). We have also observed that the other nine miRNA clusters (mir-371a/373, mir-24-2/23a, mir-99b/125a, mir-6804/6803, mir-4745/3187, mir-1227/6789, mir-6885/6790, mir-181c/181d, mir-642a/642b) are also co-localized with various RCNV regions on Chr19 (Supplementary Table S4). The CFSs are heritable specific loci on chromosomes and that exhibit an increased frequency of chromosomal breakages. We identified C19MC and six other miRNA clusters (mir-4754/10394, mir-3190/3191, mir-642a/642b, mir-6804/6803, mir-99b/125a, mir-371a/373) that are associated with FRA19A (Fig. 1C).

### Expression correlation analysis of C19MC members

**Fig. 2:**
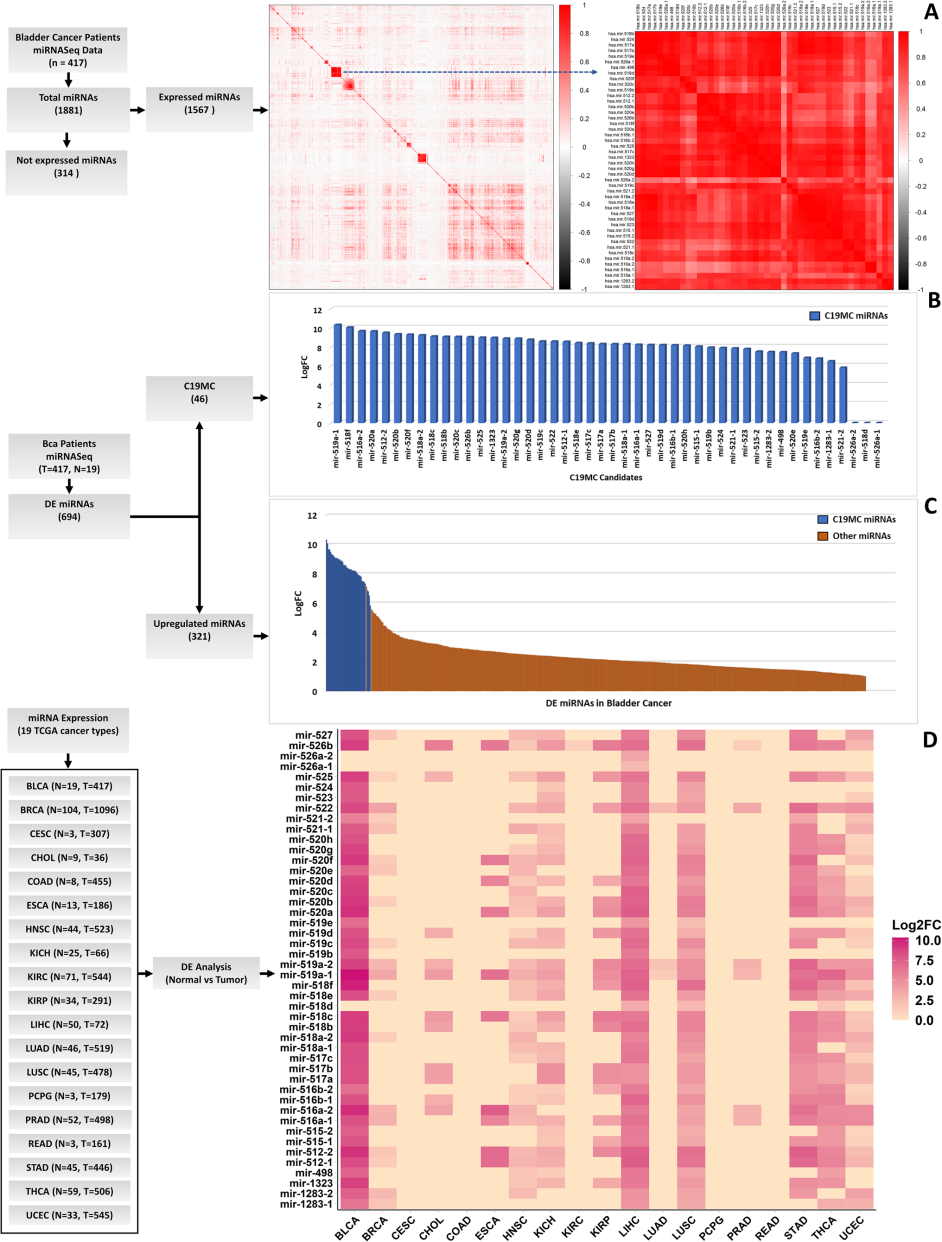
**A** Correlation analysis of all expressed miRNAs in human bladder cancer reveals that C19MC miRNA members are the most correlatively co-expressed miRNAs. **B** Differentially expressed miRNA candidates from the C19MC cluster in BCa samples. **C** The expression level of all C19MC miRNAs versus all other miRNAs expressed in BCa samples. **D** differential expression of C19MC members across TCGA pan cancer

To explore the expression pattern of C19MC miRNAs in human BCa, we used correlation analysis on the TCGA miRNASeq datasets. Among the 1881 miRNAs in the dataset, 314 were not expressed in any of the 417 BCa patients. Hence, the remaining 1567 miRNAs were examined for correlation analysis. Interestingly, we observed that 43 members of C19MC formed a tight, positively correlated, and co-expressed cluster in BCa (Fig. 2A). Correlations between C19MC members were statistically significant (*p* = < 0.05), and the heatmap plot shows a high positive correlation (deep red). There were no other miRNAs that formed clusters as dense and large as C19MC, showing that the C19MC cluster is the most prevalent co-expressed miRNA set in human BCa.

### Differential expression analysis of C19MC members

The comparative analysis between 321 upregulated DEmiRs indicates that 43 members of C19MC are highly expressed and fall under the top 50 highly expressed miRNAs in BCa patients (Fig. 2B, C). The cumulative Log2Fold expression of 43 C19MC members contributed 22% of the total upregulated miRNAs in BCa. The Log2Fold change expressions (normal vs. tumor) of C19MC members were mir-519a-1: 10.3, mir-518f: 10.03, mir-516a-2: 9.64, mir-520a: 9.62, mir-512-2: 9.47, mir-520b: 9.31, mir-520f: 9.27, mir-518a-2: 9.2, mir-518c: 9.09, mir-518b: 9.05, mir-520c: 9.04, mir-526b: 9.01, mir-525: 8.96, mir-1323: 8.94, mir-519a-2: 8.86, mir-520g: 8.85, mir-520d: 8.74, mir-519c: 8.56, mir-522: 8.56, mir-512-1: 8.52, mir-518e: 8.39, mir-517c: 8.36, mir-517a: 8.29, mir-517b: 8.27, mir-518a-1: 8.27, mir-516a-1: 8.21, mir-527: 8.19, mir-519d: 8.18, mir-516b-1: 8.16, mir-520h: 8.12, mir-515-1: 8.03, mir-519b: 7.92, mir-524: 7.87, mir-521-1: 7.83, mir-523: 7.77, mir-515-2: 7.49, mir-1283-2: 7.44, mir-498: 7.43, mir-520e: 7.3, mir-519e: 6.84, mir-516b-2: 6.76, mir-1283-1: 6.48, and mir-521-2: 5.8. However, three miRNAs (mir-526a-2, mir-518d, mir-526a-1) did not show any expression trend and belonging to the subclass of 314 miRNAs “not expressed” in BCa.

### Pan-cancer view

The entire panel of C19MC members was simultaneously activated and highly overexpressed in BCa compared with other cancer types except for mir-518d, mir-526a-1, and mir-526a-2 (Fig. 2D). Interestingly, these three members are upregulated in liver hepatocellular carcinoma (LIHC) and lung squamous cell carcinoma (LUSC). Additionally, mir-518d activation was observed in thyroid carcinoma (THCA). It is clear that there is an alternative regulator inside the cluster that controls the expression of these three miRNAs. The pan-cancer research also suggests that C19MC is activated in additional cancer types, which needs to be examined further.

### C19MC expression, RCNV and DNA methylation correlation analysis

**Fig. 3:**
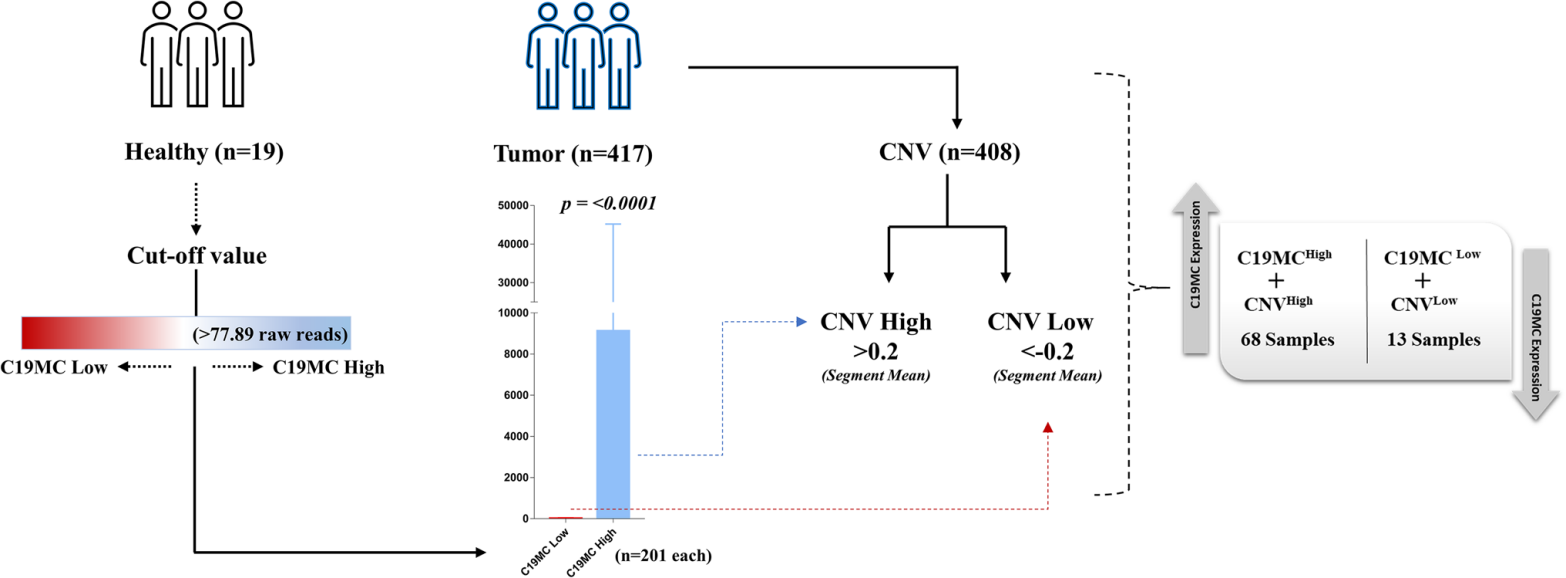
Workflow illustrating the integration of TCGA-BCa, CNV, and expression integrative analysis. A total of 68 samples were identified as having high expression with high CNV status, whereas 13 samples were identified as having low expression with low CNV status

**Fig. 4:**
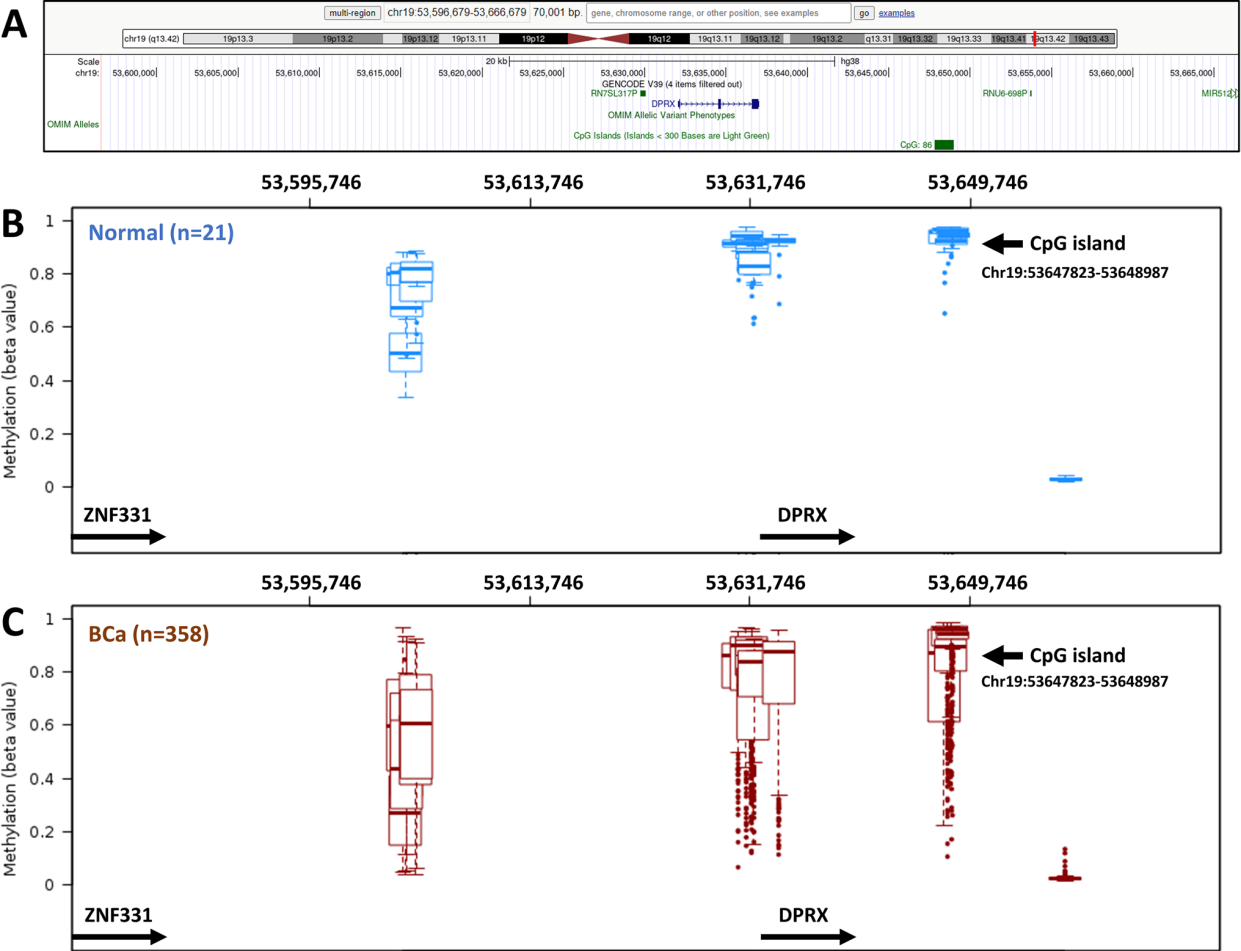
Comparative methylation status of the pre-C19MC region between normal and BCa using TCGA wanderer 450k methylation data. The genes ZNF331 and DPRX were used to locate the region. **A** The UCSC Genome Browser (hg38 build) was used to show the CpG site at pre-C19MC region. **B** Methylation at promoter CpG sites in normal samples. **C** Methylation at promoter CpG sites in BCa samples

In the TCGA-BCa cohort, we observed that C19MC co-localized with the RCNV amplified region (Chr19:53530432-54088052). For better understanding, the CNV^High^ group was correlated with the C19MC^High^ group and the CNV^Low^ group was correlated with the C19MC^Low^ group. Of the 417 samples, 68 were found to have high C19MC expression and CNV status. However, only 13 samples were determined to have low CNV and low expression status (Fig. 3). To investigate the role of epigenetic modification in the regulation of C19MC expression, we used 450k methylation array data to examine the DNA methylation pattern of ~70kb C19MC upstream region. When compared to normal samples, patients with BCa samples showed significant hypomethylation at all promoter CpG sites. Interestingly, the probes at CpG islands (Chr19:53647823-53648987) were observed as hypomethylated in a large cohort of BCa (Fig. 4). Furthermore, the CpG island probes (cg15096240, cg09065632, cg16187069, and cg00886824) were thoroughly examined, and all BCa samples were classified based on their methylation beta value (Supplementary Table S5). A closer examination revealed that all four probes within the CpG-island exhibited hypomethylation in BCa, but cg09065632 had more hypomethylation with a significant *p* value (0.0095) (Supplementary Fig. 1). The other 3 probes (cg15096240, cg16187069, and cg00886824) were not statistically significant. These findings show a weak association between DNA methylation and C19MC expression in a large BCa cohort, indicating that mechanisms other than C19MC hypomethylation are necessary for C19MC overexpression.

### Identification of transcription regulators of C19MC

**Fig. 5:**
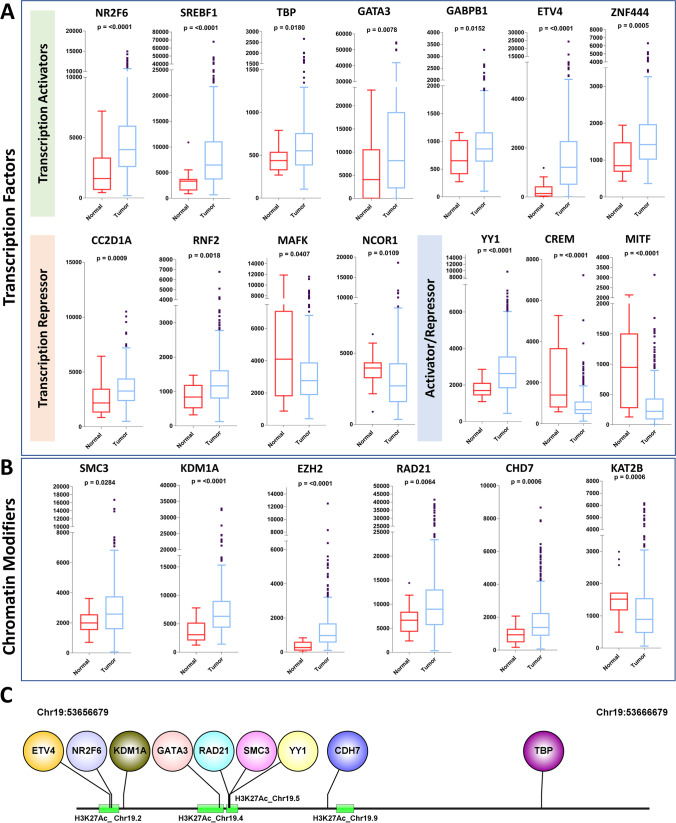
The differential expression analysis of TFs and CMs which binds at C19MC promoter site. Expression analysis among normal and BCa samples revealed **A** 7 transcription activators and **B** 5 chromatin modifiers are significantly upregulated BCa

Data analysis using the UCSC Genome Browser revealed that the region around the C19MC start site contains a series of strong and weak TF binding sites and regulatory polymorphisms (Supplementary Fig. 2). We observed that a series of H3K27Ac protein span over C19MC regions. The H3K27Ac separates active enhancers from inactive/poised enhancer elements, which are linked with increased transcription activation. To extend the study, genomic regions were categorized as pre-C19MC (Chr19:53656679-53666679, 10kb upstream), C19MC (Chr19:53666679-53762430), and post-C19MC (Chr19:53666679-53676679, 10kb downstream). The prediction of TFs and chromatin modifiers (CMs) from these regions is shown in Supplementary Fig. 3. The expression analysis revealed that seven transcription activators (*NR2F6*, *SREBF1*, *TBP*, *GATA3*, *GABPB1*, *ETV4*, and *ZNF444*) and five CMs (*SMC3*, *KDM1A*, *EZH2*, *RAD21*, and *CHD7*) interact with the C19MC promoter region (pre-C19MC) and were significantly upregulated (Fig. 5). We observed that *SMC3* and *RAD21* show strong binding to the H3K27Ac mark located on the C19MC promoter (Fig. 5C). Furthermore, *KDM1A* and *CHD7* binding sites exist very close to the H3K27Ac mark. The *SMC3* and *RAD21* are higher-order chromatin structural regulators that operate as cohesin core factors to shield chromatin. The other overexpressed transcription factor is *YY1* which belongs to the class of GLI-Kruppel zinc finger proteins and shows high binding affinity at C19MC promoter. Reports suggest that *YY1* can act as a transcriptional activator and repressor in mammalian systems (Verheul et al. 2020).

### Prediction of C19MC target genes

To examine the interactions and effects of C19MC on target genes, we used a combination of tools to uncover target gene interactions. A total of 616 target genes (286 upregulated and 330 downregulated) were identified after mapping the C19MC-target gene interaction and significant DEGs (Supplementary File 1). Each miRNA may target hundreds of genes, and a single gene can be targeted by many miRNAs, resulting in a complex interaction network. The complexity and effect of miRNAs on target genes increase when a set of targeting miRNAs belongs to the same family or a cluster. We identified the top 5 sets of genes that were targeted by the majority of the C19MC (Supplementary Fig. 4). We observed that 13 different miRNAs in the top tier of C19MC targeted the *WEE1* gene. The second most genes being targeted by 11 members of C19MC are *MAP1B*, *NACC2*, and *CRISPLD2*. Four genes *RAB3IP*, *CAVIN1*, *CFL2*, and *GATA6* rank third being targeted by 10 miRNAs. There are nine miRNAs that target *MELK*, *AURKA*, *AGMAT*, *ZNF107*, *DPYSL2*, *CCND2*, *DNAJB4*, *FBXL7*, and *KCND3* and eight miRNAs that target *APOH*, *HEPHL1*, *DRAXIN*, *DNAJB13*, *MKI67*, *ZYG11A*, *BMP8B*, *SLC28A1*, *ZNF682*, *FAAP24*, *MTHFD1L*, *PRIM1*, and *KIAA0513*, making 4^th^ and 5^th^ groups. The top five C19MC members with a large number of targets included mir-522-5p which targets a maximum of 179 genes, followed by mir-520c-3p (130), mir-520f-3p (106), mir-519d-3p (72), and mir-520a-3p (64 targets) (Supplementary Fig. 5).

### Status of C19MC targeted TS genes in BCa

**Fig. 6:**
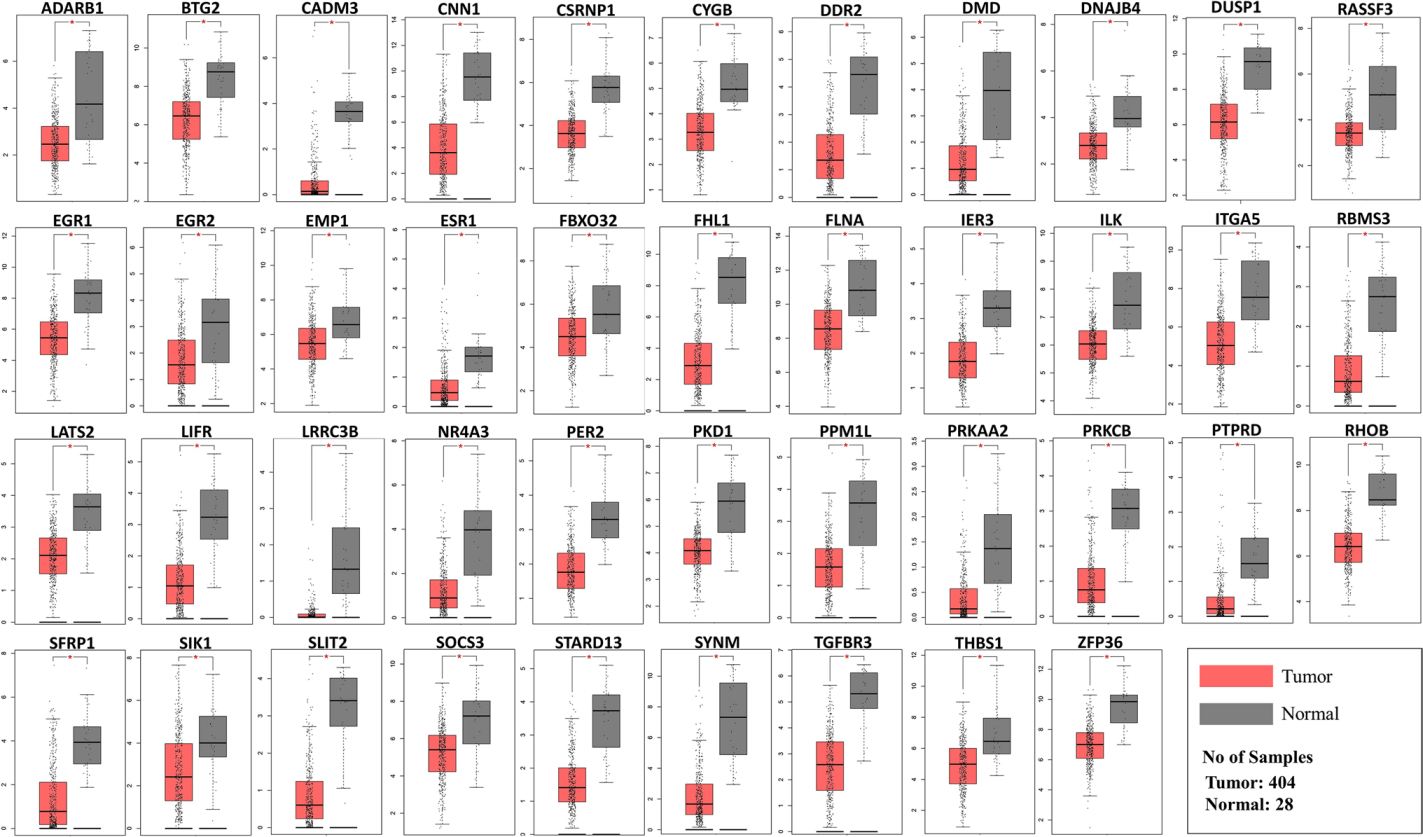
The differential expression of the 42 TS genes in normal vs. BCa tissues (*p* value < 0.01). Expression data was procured from the GEPIA, an online resource for gene expression profiling and interactive analysis

**Fig. 7:**
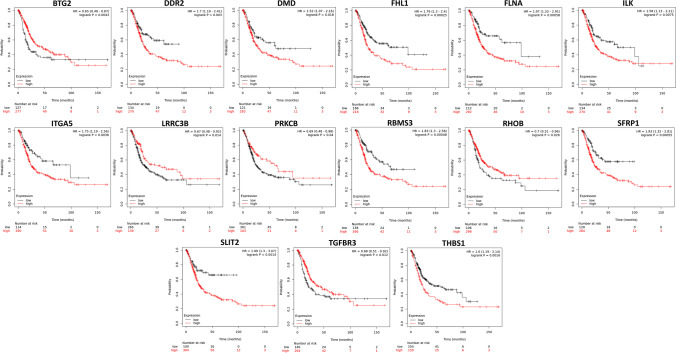
Prognostic feature analysis of C19MC-targeted tumor suppressor gene candidates in BCa patients

We found that 42 TS genes targeted by C19MC members are significantly downregulated in BCa, out of 493 TS genes predicted in BCa. (Fig. 6). Additionally, we compared the 42 TS genes to other cancer types. Tumor suppressors such as *DNAJB4*, *EMP1*, *LATS2*, *LIFR*, *SFRP1*, *CSRNP1*, *TGFBR3*, and *BTG2* have previously been reported in 11 TCGA cancer types (BLCA, BRCA, COAD, HNSC, KICH, KIRC, LUAD, LUSC, PRAD, THCA, and UCEC). The details of all TS genes are provided in Supplementary Table 7. In the TCGA-BCa cohort, we compared survival analysis and hazard ratio with populations designated as TS genes high and low risk. The Kaplan–Meier (KM) survival analysis revealed a total of 15 prognostically significant genes (Fig. 7). The overall survival rates of candidates are *BTG*: HR = 0.65, *p* = 0.0043; *DDR2*: HR = 1.7, *p* = 0.003; *DMD*: HR = 1.52, *p* = 0.018; *FHL1*: HR = 1.76, *p* = 0.00025; *FLNA*: HR = 1.97, *p* = 0.00058; *ILK*: HR = 1.58, *p* = 0.0075; *ITGA5*: HR = 1.75, *p* = 0.0038; *LRRC3B*: HR = 0.67, *p* = 0.014; *PRKCB*: HR = 0.69, *p* = 0.04; *RBMS3*: HR = 1.83, *p* = 0.00048; *RHOB*: HR = 0.7, *p* = 0.026; *SFRP1*: HR = 1.93, *p* = 0.00055; *SLIT2*: HR = 1.99, *p* = 0.0014; *TGFBR3*: HR = 0.68, *p* = 0.012; and *THBS1*: HR = 1.6, *p* = 0.0016. We found that downregulation of all 15 TS genes significantly correlated with a worse survival rate in patients with BCa. Loss of *DDR2*, *DMD*, *FHL1*, *FLNA*, *ILK*, *ITGA*, *RBMS3*, *SFRP1*, *SLIT2*, and *THBS1* expression was associated with a decreased likelihood of survival and an increased risk of sudden death in patients. According to the conclusive KM findings, the predictive model comprising members of the TS gene panel is effective at predicting the progression of cancer cells in BCa patients.

### Network construction and functional enrichment analysis

**Fig. 8:**
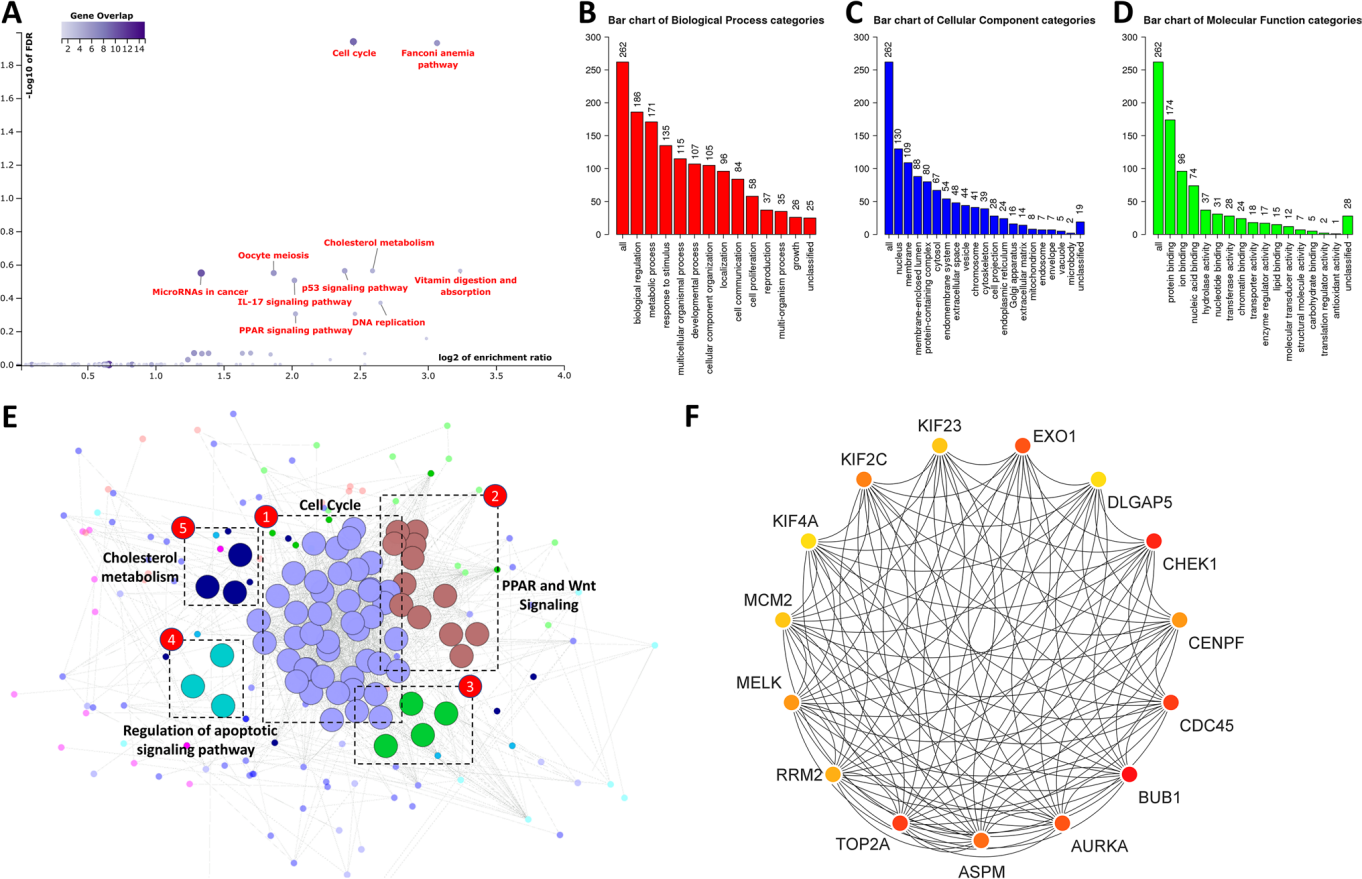
Pathway enrichment and GO analysis of C19MC target genes. **A** In the scatter diagram, y-axis represents the significant value of each enriched pathway based on the negative log10 (FDR). The x-axis represents the enrichment value of each enriched pathway based on log2 (enrichment ratio). The intensity of the color stands for the negative log10 (FDR) of each enriched pathway, as indicated on the bar on the right of scatter plot. Each dot represents a given pathway, and the size of dot showed the gene set size of each enriched pathway. Bar chart showing the number of genes that are involved in the different Gene Ontology terms as predicted by the GSEA via WebGestalt. **B** Biological process. **C** Cellular component. **D** Molecular function. **E** The significant modules in the protein-protein interaction network generated with MCODE. **F** Top 15 hub genes identified by CytoHubba

A total of 616 targeted genes were investigated in depth to identify additional genes beyond tumor suppressors that may contribute to cellular signaling and other processes in BCa. A total of 67 genes out of 616 were excluded from the subsequent study as they were either associated with CNV-deleted regions or hypermethylated regions in BCa patients (Supplementary Table 8). Furthermore, we assumed that the remaining 263 genes were downregulated as a result of C19MC upregulation. These genes were significantly linked with 10 cancer-associated pathways such as cell cycle regulation, DNA replication, oocyte meiosis, p53 signaling, Fanconi anemia pathway, IL-17 signaling, and PPAR signaling (Fig. 8A). We also identified genes that were enriched for cancer-associated miRNAs and cholesterol metabolism. These gene sets and associated BP, CC, and MF are illustrated as a bar graph (Fig. 8B–D). The protein functional network included 263 nodes (genes) and 1422 edges (interactions), with a protein-protein interaction (PPI) enrichment *p* value of <1.0*e−*16. Four significant modules were defined by MCODE, and their enriched functions are illustrated in Fig. 8E. We identified the top 15 genes (*BUB1*, *AURKA*, *MELK*, *ASPM*, *TOP2A*, *RRM2*, *MCM2*, *KIF4A*, *KIF2C*, *KIF23*, *EXO1*, *DLGAP5*, *CHEK1*, *CENPF*, and *CDC45*) based on their high node degree in the PPI network (Fig. 8F).

### Evaluation of BCa-infiltrating immune cells and the tumor microenvironment

**Fig. 9:**
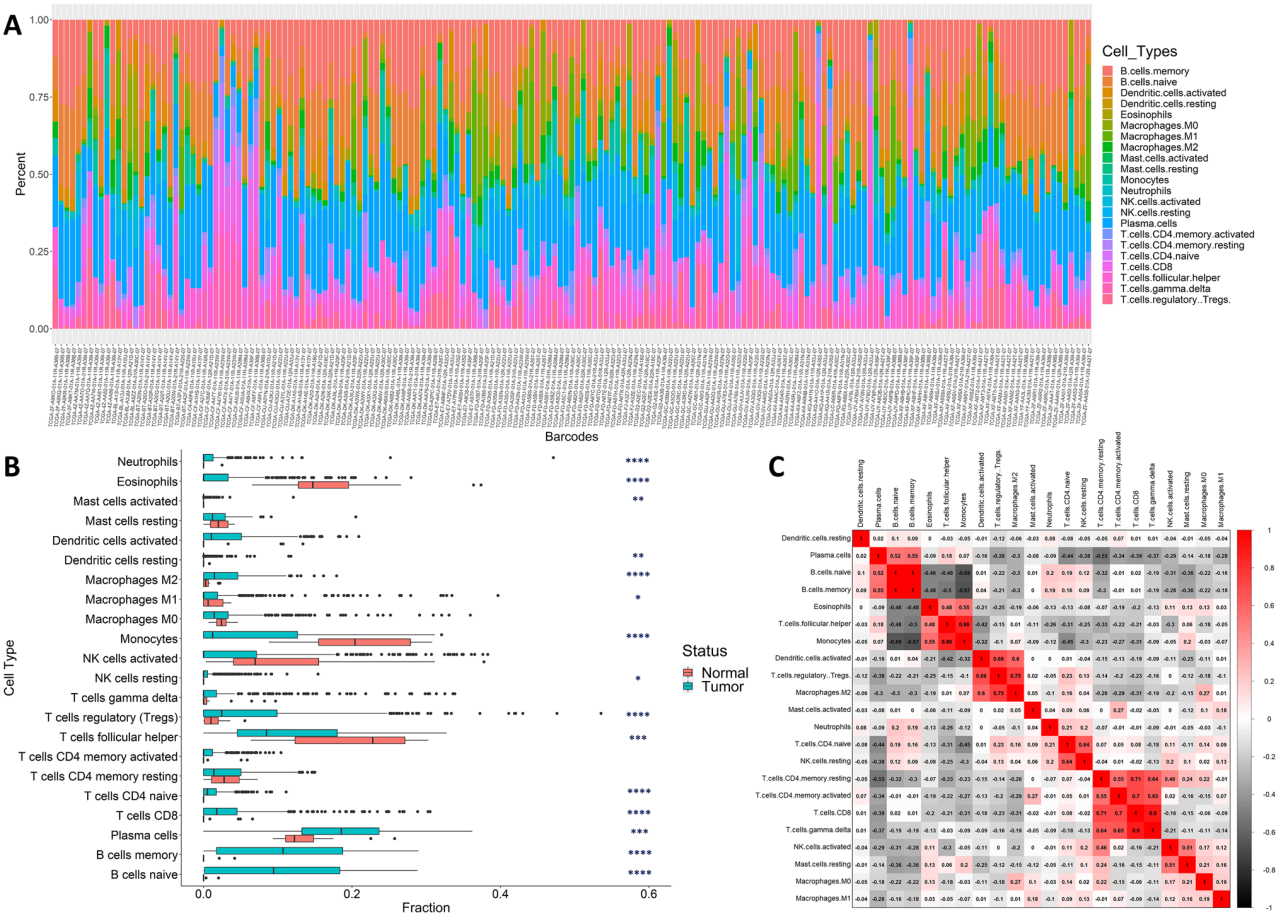
Status of immune cell infiltration in the TCGA-BCa cohort. **A** The bar chart summarizes the percentage of 22 infiltrated immune cells from BCa (*n*=182) tissues. Each color represents the type of immune cell and the length of the bar represents the relative percentage of infiltrating immune cells. **B** The compositions of tumor-infiltrating cells in the BCa and control groups are shown by the box plot (*p < 0.05; **p < 0.01; ***p < 0.001; ***p < 0.0001). **C** Correlation matrix of all 22 immune cell proportions in TCGA BCa cohort

We generated the relative immune fraction score using CIBERSORT analysis, which estimates the proportion of each immune cell type such that the total of all fractions equals 100% for a given mixed sample. A total of 182 effective BCa samples were obtained. Figure 9A shows the total proportions of tumor-infiltrating cells in BCa. The lengths of the bars in the chart indicate the levels of the immune cell populations. No significant normal samples were obtained in the immune infiltration analysis. The infiltration proportion of T cells CD4 memory resting (29.37%), plasma cells (18.14%) was the highest, and that of Mast cells activated (0.09%) was the lowest in BCa tissues (Supplementary Table 9). A box plot (Fig. 9B) depicts the difference in immune cell distribution between BCa and adjacent normal samples. Among the 22 immune cell types, the proportion of infiltrating B cells memory, naïve B cells, T cells CD8, T cells regulatory (Tregs), neutrophils, and eosinophils in BCa tissues was significantly higher than that of normal tissues. In contrast, there was no significant difference observed in the infiltration of T cells CD4 memory resting, mast cells activated, mast cells resting, dendritic cells resting, NK cells resting, and T cells CD4 memory activated compared with the normal group. The correlation finding shows that subpopulations of various tumor-infiltrating immune cells were weakly to moderately associated (Fig. 9C). These results showed that there were different infiltration patterns of immune cells invading normal and BCa tissues that could be used to distinguish BCa from adjacent controls.

## Discussion

The largest human miRNA cluster, C19MC, is reported as overexpressed in various types of cancer (Rippe et al. 2010; Strub et al. 2016; Jinesh et al. 2018; Sin-Chan et al. 2019). However, understanding their regulatory mechanisms modulating disease outcomes is still in its infancy. According to previous reports, C19MC activation is a common event in tumorigenesis, since alteration in transcriptional regulation, 19q13.4 rearrangements, genomic amplification, and methylations anomalies have been frequently observed in a variety of human neoplasia (Rippe et al. 2010; Vaira et al. 2012; Jinesh et al. 2018). Moreover, the DNA methylation status at the CpG site region approximately 17.6 kb upstream was reported to be critical in C19MC regulation (Tsai et al. 2009). In our previous study (Ware et al. 2022b), we found that C19MC was upregulated and co-localized with RCNV in BCa. Further investigation revealed that C19MC member expression was not statistically significant in patient survival analyses. Hence, we conducted an integrative multiomics analysis to gain more insights and understand the C19MC regulation in BCa.

The comparative expression analysis showed that C19MC miRNA candidates exhibited positively correlated co-expression in BCa (Fig. 2A). Among the 1567 miRNAs expressed in 417 patients, no other miRNA group formed a cluster as large and dense as C19MC. We observed a sustained 5–10-fold increase in expression for 43 C19MC candidates, which was higher than the overall expression of all other miRNAs in BCa (Fig. 2B, C). This evidence supports the hypothesis that the expression of C19MC members in BCa is regulated by the same transcriptional regulator.

The majority of dysregulated miRNA genes in tumor conditions usually span at CFS in the genome, and their dysregulated expression promotes cancer development (Calin et al. 2004). The CFSs were reported to have a significant role in generating focal copy number alterations that affect the genomic landscape of various malignancies (Glover et al. 2017). Of note, we observed that C19MC co-localized with the RCNV gain region (Chr19:53530432-54088052) in BCa patients data at the TCGA portal and that CNV-associated C19MC extends over the region of FRA19A.

Compared to the normal samples, BCa observed considerable hypomethylation in the promoter (Chr19:53596679-53666679) region of larger BCa cohort, other than 17.6 kb upstream CpG island sites (Fig. 4). The probes at CpG islands were not statistically significant, except cg09065632. Interestingly, the group-wise analysis between C19MC^High^ expression and CNV^High^ status revealed that CNVs have no major impact on C19MC expression. Nonetheless, C19MC cluster expression and RCNV gain were not mutually exclusive with contradictory evidence found in a limited number of tumors. Indeed, RCNV gain was not conclusively linked to C19MC upregulation in BCa patients. Although it is obvious that CFS plays an important role in the production of CNVs in BCa, a more complete study of the combined and independent actions of CFS and CNVs with regard to C19MC regulation in diverse BCa populations would be extremely interesting. Furthermore, hypomethylation in the pre-C19MC region may be involved in C19MC expression regulation; nonetheless, the functional relevance must be evaluated further to conclude the epigenetic contribution to BCa distribution.

Enhancers are potent regulators of gene expression patterns; however, identifying enhancer regions and their contribution to target gene expression remains challenging. H3K4me1 and H3K27Ac chromatin signatures have previously been shown to predict potential enhancers. Particularly, H3K27Ac is an important mark because it distinguishes active enhancers from poised enhancers (Creyghton et al. 2010). Interestingly, we observed a succession of strong and weak H3K27Ac marks at the C19MC start site, indicating the possibility of enhancer-mediated C19MC regulation (Supplementary Fig. 2). TFs can also act as upstream regulators of miRNAs that can promote or suppress miRNA expression. TFs along with miRNAs can influence target gene expression and form feedback or feed-forward loops (Zhang et al. 2013). Considering this, we identified seven TFs (*NR2F6*, *SREBF1*, *TBP*, *GATA3*, *GABPB1*, *ETV4*, and *ZNF444*) that appeared to occupy and bind across the promoter site of C19MC. All these TFs were found to be significantly upregulated in BCa compared to the normal samples (Fig. 5). Although *REST* is the sole transcription repressor reported to have binding sites across C19MC, its expression shows no discernible change in tumor conditions (Chen et al. 1998). *RAD21* and *SMC3* are higher-order chromatin structural regulators that operate as cohesion core factors to insulate chromatin (Zuin et al. 2014). The TFs and CMs are the key regulatory factors that frequently collaborate to accomplish precise genomic control (Zhang et al. 2018). In support of this view, we identified that the majority of anticipated TF binding sites are located exactly on H3K27Ac marks (Fig. 5). Our findings provide new information on the dynamic regulation of C19MC through the combined control of TFs and enhancers at the C19MC start site, implying that all miRNA candidates in this cluster are under the same transcription control.

The oncogenic role of upregulated C19MC members mir-522-5p, mir-520c-3p, mir-520f-3p, mir-519d-3p, and mir-520a-3p has been extensively reported in various malignancies (Huang et al. 2008; Zhang et al. 2017; Zhou et al. 2016; Sun et al. 2020). We identified these five members, which also showed the highest number of gene targets. Similarly, the *WEE1* gene is targeted by 13 C19MC members, topping the list of targeted genes. Contrary to expectations, *WEE1* is significantly upregulated in BCa. Inhibition of WEE1 has been shown to target cell cycle checkpoints to overcome cisplatin resistance (Zheng et al. 2017). While our data suggest that *WEE1* and the targets of C19MC members can be a viable therapeutic target in BCa, further research into the sponges responsible for redirecting the C19MC members is warranted.

There were 42 members of C19MC-targeted tumor suppressor genes identified by mapping against a comprehensive tumor suppressor database. Of note, the downregulation of 15 genes was established to have a significant correlation with the overall survival rate of BCa patients. Researchers have shown that a lack of *PTEN* expression is associated with aggressive BCa. PTEN and p53 are important for inducing the tumor suppressor gene *BTG2* (Lee et al. 2012; Tsui et al. 2018). Our investigation emphasizes the importance of four top ranked members of C19MC, miR-522-5p, miR-519d-3p, miR-526b-3p, and miR-520a-5p, which may have critical roles in the regulation of *BTG2* in BCa. The TGF receptor III (*TGFBR3*) participates in the TGF-mediated signaling pathway by enhancing TGF binding to *TGFBR3*, which is responsible for a variety of critical cellular processes, including cell proliferation, adhesion, and migration (Liu et al. 2013). Krüppel-like factors (*KLFs*) have recently been reported to suppress TGF-type III receptor (*TGFBR3*) expression by binding to its promoter region and lowering transcriptional activity in BCa (Chen et al. 2022). Similarly, we found that miR-518a-3p targets *TGFBR3*, suggesting that it may play a substantial role in the regulation of this gene. Leucine-rich repeat (LRR)-containing 3B (*LRRC3B*) inhibits proliferation and invasion by targeting β-catenin, cyclin D1, and c-Myc in bladder cancer cells; nevertheless, its expression has been observed to be substantially downregulated in BCa (Zhao et al. 2017). The miR-520f-3p, a C19MC member of the third tier, was identified to target LRRC3B. Integrin-linked kinase (*ILK*) is a crucial signaling factor in epithelial-mesenchymal (*EMT*) transition and is a major element of the cadherin switch (Gil et al. 2016). We found that *ILK* is targeted by miR-520a-5p and was significantly downregulated in BCa. In contrast, the upregulation of Cadherin-1 (*CDH1*) in BCa is observed. This suggests that there is an inverse correlation between *ILK* and *CDH1*. Research into the coordinated regulation of *ILK* by miR-520a-5p is needed.

Further investigation revealed that miR-518c-5p, miR-520c-3p, and miR-520d-5p target the *ITGA5*, *FHL1*, and *SLIT2* genes, respectively, whereas miR-522-5p targets *FLNA* and *SFRP1*. The miR-520h and miR-520g-3p target *DMD*, whereas miR-522-5p, miR-518c-5p, miR-526b-5p, and miR-516a-5p targeted *THBS1* and *RBMS3*, separately. *PRKCB* is the target of four distinct microRNAs: miR-519d-3p, miR-526b-3p, miR-520a-5p, and miR-525-5p. Log-rank test-based Kaplan–Meier survival analysis showed that all the above-mentioned genes were significantly correlated with the overall survival rate of BCa patients. Their downregulation is strongly associated with the worst prognosis in BCa patients. Our research suggests that these C19MC-targeted TS gene profiles may be useful as prognostic indicators for BCa and in the treatment of cancer. Research into the abnormal regulation of the C19MC-TS gene has the potential to simplify therapy and increase cancer cell sensitivity to drugs.

Recent findings indicate that infiltrating immune and stromal cells are critical components of the BCa tumor microenvironment (TME) and have a substantial impact on the progression and outcome of malignancy (Zhang et al. 2020). In terms of chemotherapy and immunotherapy, the composition of tumor-infiltrating immune cells can serve as biomarkers for predicting response to treatment and survival in distinct patient subgroups. Using RNAseq datasets, we estimated the immune cell infiltration pattern in BCa-TME and acquired a total of 182 significant (*p* value <0.05) tumor samples. The proportion of T cells CD4 memory resting (29.37 %) and plasma cells (18.14 %) infiltration was the highest in these samples. The proportion of infiltrating B cells memory, naïve B cells, T cells CD8, T cells regulatory (Tregs), neutrophils, and eosinophils in BCa tissues was found to be considerably greater than that in normal tissues (Fig. 9). These distinct immune infiltration patterns of tumor-infiltrating cells are important determinants of BCa prognosis.

We also attempted to gain a better understanding of the downstream regulatory pathway by employing C19MC and target genes. According to GSEA and MCODE analysis, C19MC regulates a diverse group of genes involved in numerous cell cycle regulations and tumors are commonly recognized to have disruption of the cell cycle (Fig. 8). Several routes from the identified pathways and gene list have already been recognized and linked to BCa development. The relationship between C19MC and target genes needs to be thoroughly investigated at all levels, including tumor grades, clinical stages, diagnostic subtypes, and therapeutic response groups.

## Conclusion

In summary, we performed integrated multiomics data analytics to explore the expression pattern and regulatory mechanism of the largest human miRNA cluster (C19MC). We identified that 43 members of the C19MC are under the same transcriptional control, co-expressed, and upregulated in BCa. Although C19MC is co-localized with the recurrent copy number gain region in BCa, C19MC is not conclusively regulated by CNV. The C19MC promoter regions were significantly hypomethylated at various CpG sites except the probes at CpG island (upstream ~17.6 kb) in large cohort of BCa compared with normal. As a result, hypomethylation in the pre-C19MC region (other than CpG islands) may contribute to C19MC expression regulation, which requires additional investigation at the patent level. Interestingly, the study identified seven TFs as key players capable of activating the C19MC in BCa patients. The C19MC-targeted TS genes have an important role in BCa and significantly impact patient survival and diagnosis. Our findings contribute to a better knowledge of C19MC regulation in BCa, as well as new perspectives on C19MC as a therapeutic target. More wet-lab studies are needed to validate these findings.

## Limitations and future prospective

The findings of this study can aid in identifying potential targets for intervention strategies in BCa patients. However, to ensure the credibility of utilizing these targets, studies specific to the population must be conducted. The key genes identified with therapeutic significance can be further validated for specific populations. Currently, there is no literature on the investigation of the largest human miRNA cluster (C19MC) and its regulation in BCa. To gain a more comprehensive understanding of the regulatory networks controlled by C19MC, it is important to examine the distinct expression patterns of the C19MC members. This requires conducting integrative multiomics investigations across various cancer types and cohorts specific to different populations.

## Supplementary information

ESM 1 (DOCX 3694 kb)

ESM 2 (XLSX 305 kb)

## Data Availability

All data generated or analyzed during this study are included in this published article and its supplementary information files.

## References

[CR1] Bader GD, Hogue CW (2003). An automated method for finding molecular complexes in large protein interaction networks. BMC Bioinformatics.

[CR2] Bentwich I, Avniel A, Karov Y, Aharonov R, Gilad S, Barad O, Barzilai A, Einat P, Einav U, Meiri E, Sharon E, Spector Y, Bentwich Z (2005). Identification of hundreds of conserved and nonconserved human microRNAs. Nat Genet.

[CR3] Calin GA, Sevignani C, Dumitru CD, Hyslop T, Noch E, Yendamuri S, Shimizu M, Rattan S, Bullrich F, Negrini M, Croce CM (2004). Human microRNA genes are frequently located at fragile sites and genomic regions involved in cancers. Proc Natl Acad Sci USA.

[CR4] Chang Y, Jin H, Li H, Ma J, Zheng Z, Sun B, Lyu Y, Lin M, Zhao H, Shen L, Zhang R, Wu S, Lin W, Lu Y, Xie Q, Zhang G, Huang X, Huang H (2020). MiRNA-516a promotes bladder cancer metastasis by inhibiting MMP9 protein degradation via the AKT/FOXO3A/SMURF1 axis. Clin Transl Med.

[CR5] Charpentier M, Gutierrez C, Guillaudeux T, Verhoest G, Pedeux R (2021). Noninvasive urine-based tests to diagnose or detect recurrence of bladder cancer. Cancers (Basel).

[CR6] Chen J, Bardes EE, Aronow BJ, Jegga AG (2009). ToppGene suite for gene list enrichment analysis and candidate gene prioritization. Nucleic Acids Res.

[CR7] Chen X, Wang P, Ou T, Li J (2022). KLF16 Downregulates the expression of tumor suppressor gene TGFBR3 to promote bladder cancer proliferation and migration. Cancer Manag Res.

[CR8] Chen ZF, Paquette AJ, Anderson DJ (1998). NRSF/REST is required in vivo for repression of multiple neuronal target genes during embryogenesis. Nat Genet.

[CR9] Chin CH, Chen SH, Wu HH, Ho CW, Ko MT, Lin CY (2014). cytoHubba: identifying hub objects and sub-networks from complex interactome. BMC Syst Biol.

[CR10] Chowdhury SG, Ray R, Karmakar P (2023). Exosomal miRNAs-a diagnostic biomarker acting as a guiding light in the diagnosis of prostate cancer. Funct Integr Genomics.

[CR11] Colaprico A, Silva TC, Olsen C, Garofano L, Cava C, Garolini D, Sabedot TS, Malta TM, Pagnotta SM, Castiglioni I, Ceccarelli M, Bontempi G, Noushmehr H (2016). TCGAbiolinks: an R/Bioconductor package for integrative analysis of TCGA data. Nucleic Acids Res.

[CR12] Creyghton MP, Cheng AW, Welstead GG, Kooistra T, Carey BW, Steine EJ, Hanna J, Lodato MA, Frampton GM, Sharp PA, Boyer LA, Young RA, Jaenisch R (2010). Histone H3K27ac separates active from poised enhancers and predicts developmental state. Proc Natl Acad Sci USA.

[CR13] Díez-Villanueva A, Mallona I, Peinado MA (2015). Wanderer, an interactive viewer to explore DNA methylation and gene expression data in human cancer. Epigenetics Chromatin.

[CR14] Ding W, Chen J, Feng G, Chen G, Wu J, Guo Y, Ni X, Shi T (2020). DNMIVD: DNA methylation interactive visualization database. Nucleic Acids Res.

[CR15] Gil D, Ciołczyk-Wierzbicka D, Dulińska-Litewka J, Laidler P (2016). Integrin-linked kinase regulates cadherin switch in bladder cancer. Tumour Biol.

[CR16] Glover TW, Wilson TE, Arlt MF (2017). Fragile sites in cancer: more than meets the eye. Nature Rev Cancer.

[CR17] Hu H, Miao YR, Jia LH, Yu QY, Zhang Q, Guo AY (2019). AnimalTFDB 3.0: a comprehensive resource for annotation and prediction of animal transcription factors. Nucleic Acids Res.

[CR18] Huang Q, Gumireddy K, Schrier M, le Sage C, Nagel R, Nair S, Egan DA, Li A, Huang G, Klein-Szanto AJ, Gimotty PA, Katsaros D, Coukos G, Zhang L, Puré E, Agami R (2008). The microRNAs miR-373 and miR-520c promote tumour invasion and metastasis. Nat Cell Biol.

[CR19] Jinesh GG, Flores ER, Brohl AS (2018). Chromosome 19 miRNA cluster and CEBPB expression specifically mark and potentially drive triple negative breast cancers. PloS One.

[CR20] Kent WJ, Sugnet CW, Furey TS, Roskin KM, Pringle TH, Zahler AM, Haussler D (2002). The human genome browser at UCSC. Genome Res.

[CR21] Kleinman CL, Gerges N, Papillon-Cavanagh S, Sin-Chan P, Pramatarova A, Quang DA, Adoue V, Busche S, Caron M, Djambazian H, Bemmo A, Fontebasso AM, Spence T, Schwartzentruber J, Albrecht S, Hauser P, Garami M, Klekner A, Bognar L, Montes JL, Staffa A, Montpetit A, Berube P, Zakrzewska M, Zakrzewski K, Liberski PP, Dong Z, Siegel PM, Duchaine T, Perotti C, Fleming A, Faury D, Remke M, Gallo M, Dirks P, Taylor MD, Sladek R, Pastinen T, Chan JA, Huang A, Majewski J, Jabado N (2014). Fusion of TTYH1 with the C19MC microRNA cluster drives expression of a brain-specific DNMT3B isoform in the embryonal brain tumor ETMR. Nat Genet.

[CR22] Kozomara A, Birgaoanu M, Griffiths-Jones S (2019). miRBase: from microRNA sequences to function. Nucleic Acids Res.

[CR23] Kumar R, Nagpal G, Kumar V, Usmani SS, Agrawal P, Raghava GPS (2019). HumCFS: a database of fragile sites in human chromosomes. BMC Genomics.

[CR24] Lambo S, von Hoff K, Korshunov A, Pfister SM, Kool M (2020). ETMR: a tumor entity in its infancy. Acta Neuropathol.

[CR25] Lee H, Choi SK, Ro JY (2012). Overexpression of DJ-1 and HSP90α, and loss of PTEN associated with invasive urothelial carcinoma of urinary bladder: possible prognostic markers. Oncol Lett.

[CR26] Liao Y, Wang J, Jaehnig EJ, Shi Z, Zhang B (2019). WebGestalt 2019: gene set analysis toolkit with revamped UIs and APIs. Nucleic Acids Res.

[CR27] Lin S, Cheung WK, Chen S, Lu G, Wang Z, Xie D, Li K, Lin MC, Kung HF (2010). Computational identification and characterization of primate-specific microRNAs in human genome. Comput Biol Chem.

[CR28] Liu XL, Xue BX, Lei Z, Yang DR, Zhang QC, Shan YX, Zhang HT (2013). TGFBR3 co-downregulated with GATA3 is associated with methylation of the GATA3 gene in bladder urothelial carcinoma. Anat Rec (Hoboken).

[CR29] Loganathan T, Doss CGP (2023). Non-coding RNAs in human health and disease: potential function as biomarkers and therapeutic targets. Funct Integr Genomics.

[CR30] Nagy Á, Munkácsy G, Győrffy B (2021). Pancancer survival analysis of cancer hallmark genes. Sci Rep.

[CR31] Newman AM, Liu CL, Green MR, Gentles AJ, Feng W, Xu Y, Hoang CD, Diehn M, Alizadeh AA (2015). Robust enumeration of cell subsets from tissue expression profiles. Nat Methods.

[CR32] Nguyen PN, Huang CJ, Sugii S, Cheong SK, Choo KB (2017). Selective activation of miRNAs of the primate-specific chromosome 19 miRNA cluster (C19MC) in cancer and stem cells and possible contribution to regulation of apoptosis. J Biomed Sci.

[CR33] Rippe V, Dittberner L, Lorenz VN, Drieschner N, Nimzyk R, Sendt W, Junker K, Belge G, Bullerdiek J (2010). The two stem cell microRNA gene clusters C19MC and miR-371-3 are activated by specific chromosomal rearrangements in a subgroup of thyroid adenomas. PloS One.

[CR34] Ru Y, Kechris KJ, Tabakoff B, Hoffman P, Radcliffe RA, Bowler R, Mahaffey S, Rossi S, Calin GA, Bemis L, Theodorescu D (2014). The multiMiR R package and database: integration of microRNA-target interactions along with their disease and drug associations. Nucleic Acids Res.

[CR35] Sin-Chan P, Mumal I, Suwal T, Ho B, Fan X, Singh I, Du Y, Lu M, Patel N, Torchia J, Popovski D, Fouladi M, Guilhamon P, Hansford JR, Leary S, Hoffman LM, Mulcahy Levy JM, Lassaletta A, Solano-Paez P, Rivas E, Reddy A, Gillespie GY, Gupta N, van Meter TE, Nakamura H, Wong TT, Ra YS, Kim SK, Massimi L, Grundy RG, Fangusaro J, Johnston D, Chan J, Lafay-Cousin L, Hwang EI, Wang Y, Catchpoole D, Michaud J, Ellezam B, Ramanujachar R, Lindsay H, Taylor MD, Hawkins CE, Bouffet E, Jabado N, Singh SK, Kleinman CL, Barsyte-Lovejoy D, Li XN, Dirks PB, Lin CY, Mack SC, Rich JN, Huang A (2019). A C19MC-LIN28A-MYCN oncogenic circuit driven by hijacked super-enhancers is a distinct therapeutic vulnerability in ETMRs: a lethal brain tumor. Cancer Cell.

[CR36] Strub GM, Kirsh AL, Whipple ME, Kuo WP, Keller RB, Kapur RP, Majesky MW, Perkins JA (2016). Endothelial and circulating C19MC microRNAs are biomarkers of infantile hemangioma. JCI Insight.

[CR37] Su H, Jiang H, Tao T, Kang X, Zhang X, Kang D, Li S, Li C, Wang H, Yang Z, Zhang J, Li C (2019). Hope and challenge: precision medicine in bladder cancer. Cancer Med.

[CR38] Subramanyam D, Lamouille S, Judson RL, Liu JY, Bucay N, Derynck R, Blelloch R (2011). Multiple targets of miR-302 and miR-372 promote reprogramming of human fibroblasts to induced pluripotent stem cells. Nat Biotechnol.

[CR39] Sun MX, An Q, Chen LM, Guo L (2020). MIR-520f regulated itch expression and promoted cell proliferation in human melanoma cells. Dose Response.

[CR40] Sung H, Ferlay J, Siegel RL, Laversanne M, Soerjomataram I, Jemal A, Bray F (2021). Global Cancer Statistics 2020: GLOBOCAN estimates of incidence and mortality worldwide for 36 cancers in 185 countries. CA Cancer J Clin.

[CR41] Syeda ZA, Langden SSS, Munkhzul C, Lee M, Song SJ (2020). Regulatory mechanism of microRNA expression in cancer. Int J Mol Sci.

[CR42] Tang Z, Li C, Kang B, Gao G, Li C, Zhang Z (2017). GEPIA: a web server for cancer and normal gene expression profiling and interactive analyses. Nucleic Acids Res.

[CR43] Theocharidis A, van Dongen S, Enright AJ, Freeman TC (2009). Network visualization and analysis of gene expression data using BioLayout Express(3D). Nat Protoc.

[CR44] Tran L, Xiao JF, Agarwal N, Duex JE, Theodorescu D (2021). Advances in bladder cancer biology and therapy. Nat Rev Cancer.

[CR45] Tsai KW, Kao HW, Chen HC, Chen SJ, Lin WC (2009). Epigenetic control of the expression of a primate-specific microRNA cluster in human cancer cells. Epigenetics.

[CR46] Tsui KH, Chiang KC, Lin YH, Chang KS, Feng TH, Juang HH (2018). BTG2 is a tumor suppressor gene upregulated by p53 and PTEN in human bladder carcinoma cells. Cancer Med.

[CR47] Vaira V, Elli F, Forno I, Guarnieri V, Verdelli C, Ferrero S, Scillitani A, Vicentini L, Cetani F, Mantovani G, Spada A, Bosari S, Corbetta S (2012). The microRNA cluster C19MC is deregulated in parathyroid tumours. J Mol Endocrinol.

[CR48] Verheul TCJ, van Hijfte L, Perenthaler E, Barakat TS (2020). The why of YY1: mechanisms of transcriptional regulation by yin yang 1. Front Cell Dev Biol.

[CR49] Ware AP, Satyamoorthy K, Paul B (2022). CmirC: an integrated database of clustered miRNAs co-localized with copy number variations in cancer. Funct Integr Genomics.

[CR50] Ware AP, Kabekkodu SP, Chawla A, Paul B, Satyamoorthy K (2022). Diagnostic and prognostic potential clustered miRNAs in bladder cancer. 3 Biotech.

[CR51] Yang X, Zhang B (2023). A review on CRISPR/Cas: a versatile tool for cancer screening, diagnosis, and clinic treatment. Funct Integr Genomics.

[CR52] Yoshitomi T, Kawakami K, Enokida H, Chiyomaru T, Kagara I, Tatarano S, Yoshino H, Arimura H, Nishiyama K, Seki N, Nakagawa M (2011). Restoration of miR-517a expression induces cell apoptosis in bladder cancer cell lines. Oncol Rep.

[CR53] Zhang HM, Kuang S, Xiong X, Gao T, Liu C, Guo AY (2013). Transcription factor and microRNA co-regulatory loops: important regulatory motifs in biological processes and diseases. Brief Bioinform.

[CR54] Zhang L, Xue G, Liu J, Li Q, Wang Y (2018). Revealing transcription factor and histone modification co-localization and dynamics across cell lines by integrating ChIP-seq and RNA-seq data. BMC Genomics.

[CR55] Zhang R, Liu R, Liu C, Niu Y, Zhang J, Guo B, Zhang CY, Li J, Yang J, Chen X (2017). A novel role for MiR-520a-3p in regulating EGFR expression in colorectal cancer. Cell Physiol Biochem.

[CR56] Zhang Y, Ou DH, Zhuang DW, Zheng ZF, Lin ME (2020). In silico analysis of the immune microenvironment in bladder cancer. BMC Cancer.

[CR57] Zhao M, Sun J, Zhao Z (2013). TSGene: a web resource for tumor suppressor genes. Nucleic Acids Res.

[CR58] Zhao Z, Cheng H, Li Y, Liu H, Zi H, Li X, Hou J (2017). LRRC3B inhibits the proliferation and invasion of bladder cancer cells. Int J Clin Exp Pathol.

[CR59] Zheng H, Shao F, Martin S, Xu X, Deng CX (2017). WEE1 inhibition targets cell cycle checkpoints for triple negative breast cancers to overcome cisplatin resistance. Sci Rep.

[CR60] Zhou JY, Zheng SR, Liu J, Shi R, Yu HL, Wei M (2016). MiR-519d facilitates the progression and metastasis of cervical cancer through direct targeting Smad7. Cancer Cell Int.

[CR61] Zuin J, Dixon JR, van der Reijden MI, Ye Z, Kolovos P, Brouwer RW, van de Corput MP, van de Werken HJ, Knoch TA, van IJcken WF, Grosveld FG, Ren B, Wendt KS (2014). Cohesin and CTCF differentially affect chromatin architecture and gene expression in human cells. Proc Natl Acad Sci USA.

