# Supplementary material for: Integrated multiomics analysis of chromosome 19 miRNA cluster in bladder cancer
Source: Funct Integr Genomics. 2023 Aug 5;23(3):266. doi: 10.1007/s10142-023-01191-0 (PMC10404189; doi:10.1007/s10142-023-01191-0)
Supplement: Supplementary file 1 — (DOCX 3694 kb) [file 10142_2023_1191_MOESM1_ESM.docx]

**Integrated Multi-Omics Analysis of Chromosome 19 miRNA Cluster in Bladder Cancer**

Akshay Pramod Ware^1^, Kapaettu Satyamoorthy^2^, and Bobby Paul^1*^

^1^Department of Bioinformatics, Manipal School of Life Sciences, Manipal Academy of Higher Education, Manipal – 576104, Karnataka, India.

^2^Department of Cell and Molecular Biology, Manipal School of Life Sciences, Manipal Academy of Higher Education, Manipal – 576104, Karnataka, India.

***Correspondence to:** bobby.paul@manipal.edu

**Supplementary Information**

All supplementary tables are combined in a single excel file.

Table titles and captions are listed below.

**Supplementary Table S1.** Differentially expressed miRNAs in BCa compared with Normal.

**Supplementary Table S2.** Differentially expressed genes in BCa compared with Normal.

**Supplementary Table S3.** List of 167 (116 amplified, and 51 deleted) significant CNV aberrations detected by GAIA analysis in on chr19 in BCa.

**Supplementary Table S4.** Details of miRNA clusters residing on recurrent CNV regions on chr19.

**Supplementary Table S5.** Methylation beta value of 4 probes mapped at CpG island (17.5 kb upstream of C19MC).

**Supplementary Table S6.** C19MC targeted genes and their expression in BCa.

**Supplementary Table S7.** Mapping of 42 C19MC target (Tumor suppressors) across 11 types of cancers.

**Supplementary Table S8.** Details of CNV and DNA promoter methylation information of C19MC targeted genes.

**Supplementary Table S9.** Details of immune cell infiltration analysis obtained from CIBERSORT algorithm.

**Supplementary Figures**


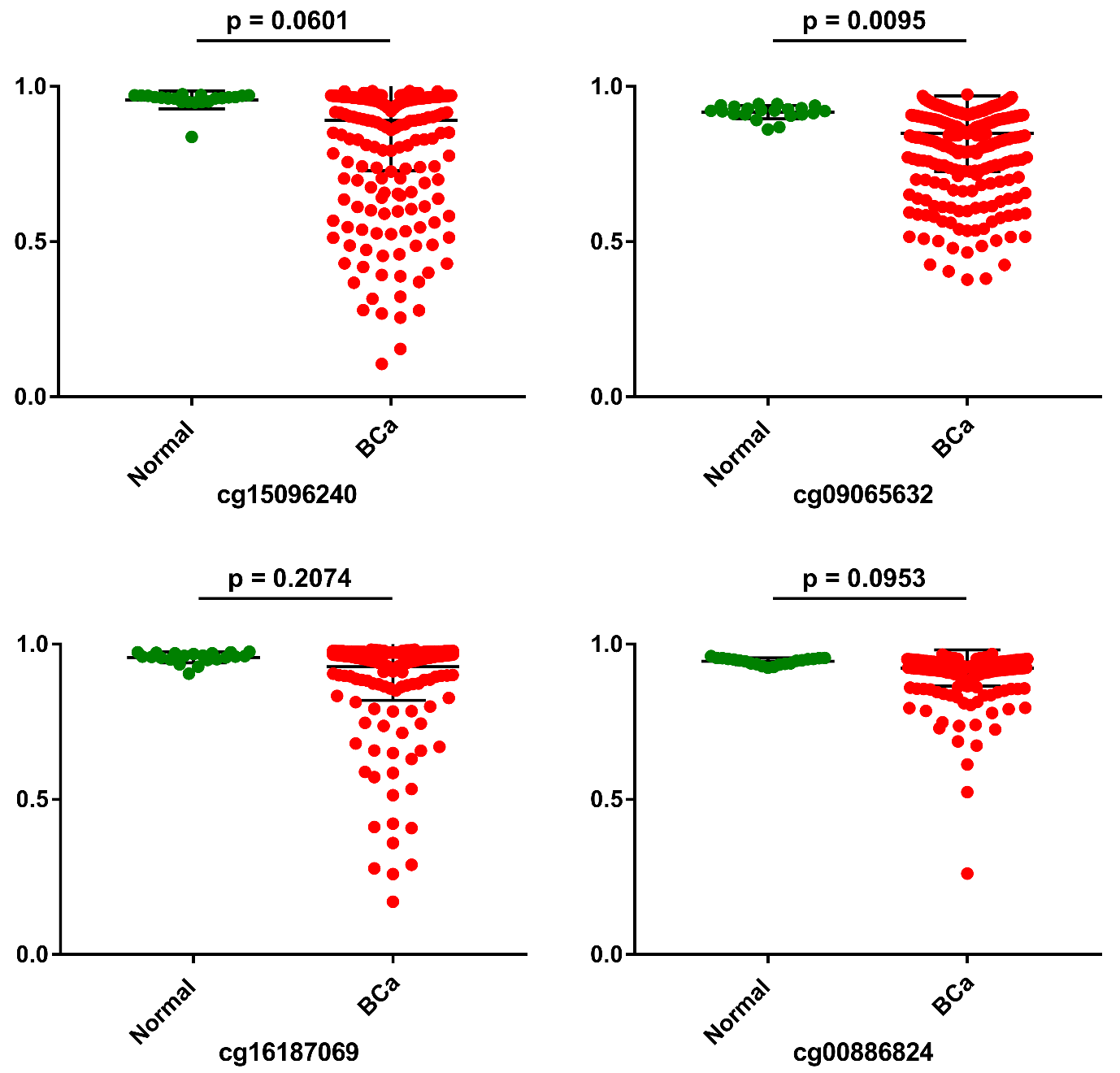


**Supplementary Fig. 1** Comparative methylation of probes at CpG-island region between normal and BCa using TCGA 450k methylation data.


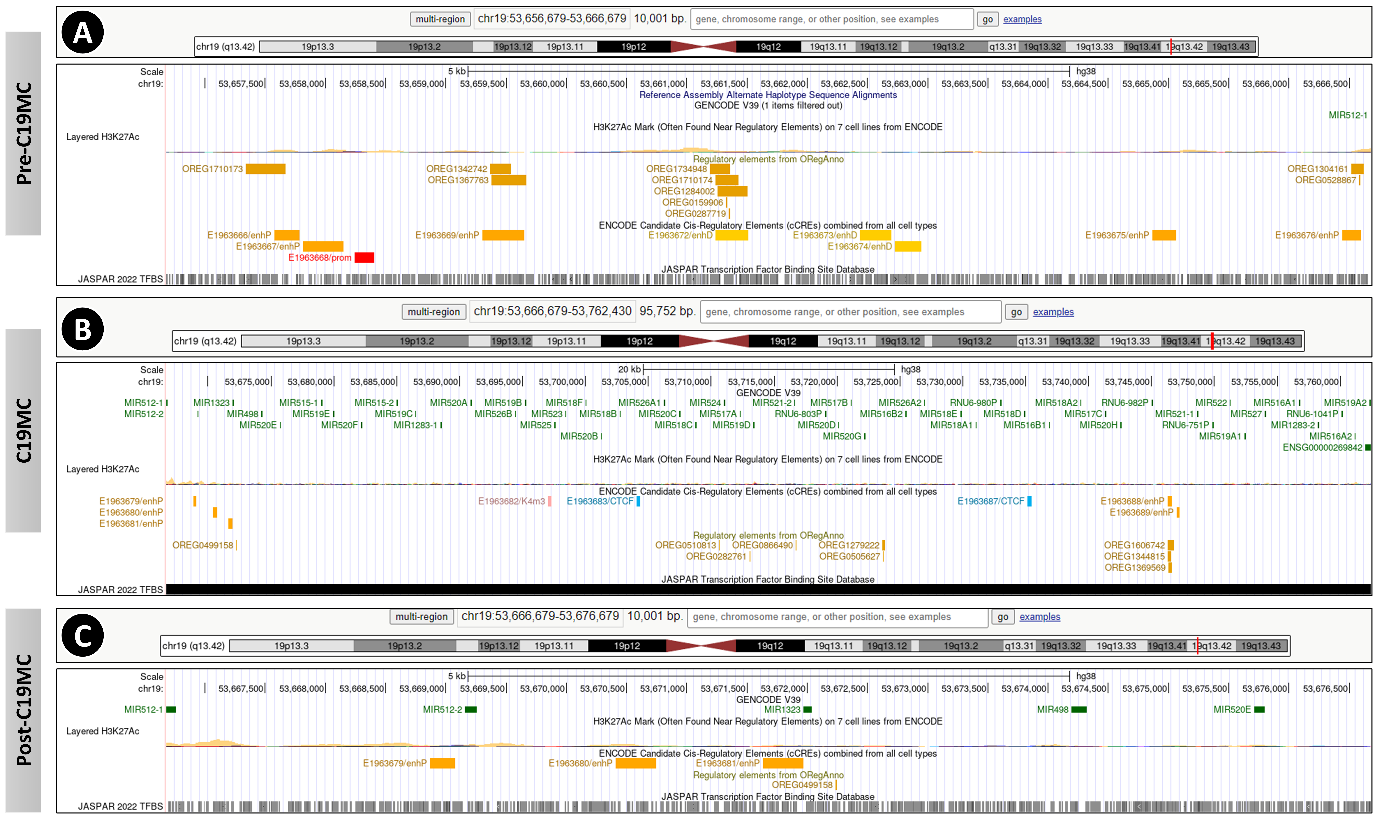


**Supplementary Fig. 2** Identification of regulatory elements in the C19MC region using the UCSC Genome Browser. UCSC Genome Browser tracks displays information about the H3K27Ac markers, enhancers, and promoter regions in the **A)** pre-C19MC, **B)** complete C19MC and **C)** post-C19MC regions.

**
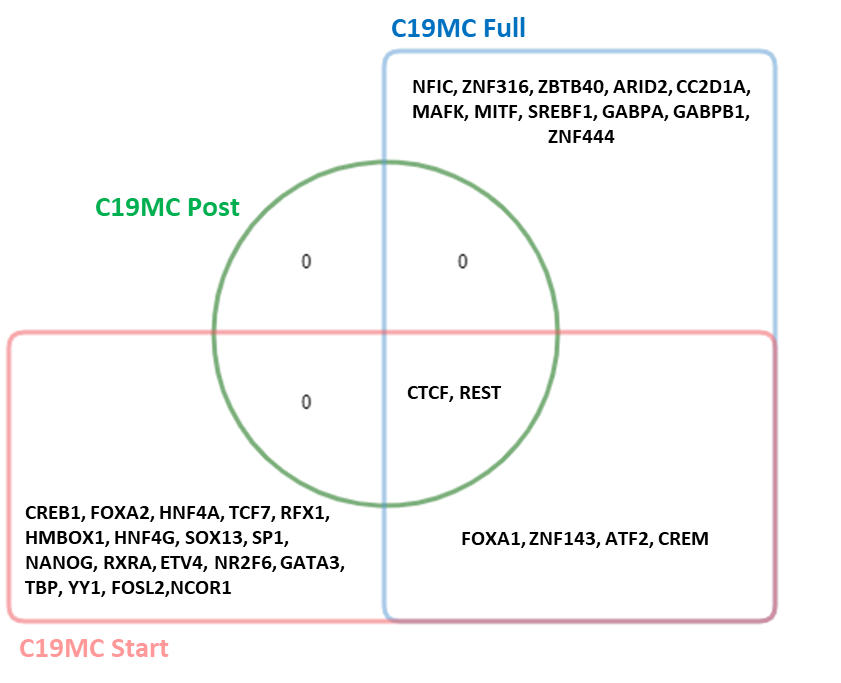
**

**Supplementary Fig. 3** Venn diagram representing the unique and common TFs targeting at three C19MC regions (pre-C19MC, full C19MC and post-C19MC)


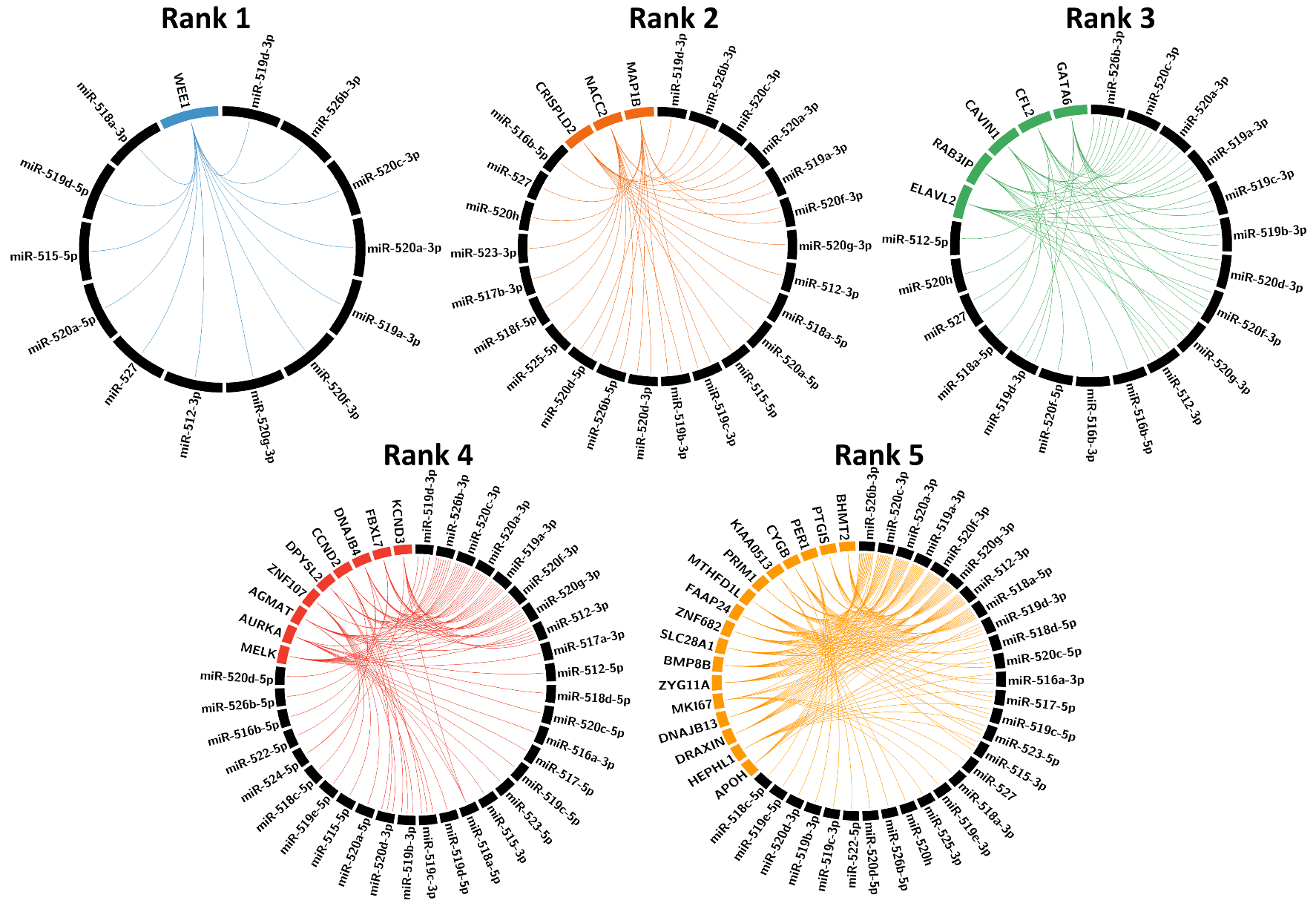


**Supplementary Fig. 4** Interaction network of C19MC members and their target genes. High confidence miRNA target genes were predicated with the help of multiMiR R/Bioconductor package. Target genes were prioritised according to the number of individual miRNAs from the C19MC that had targeted them. The miRNA-mRNA regulatory networks were visualized by using Circos tool.


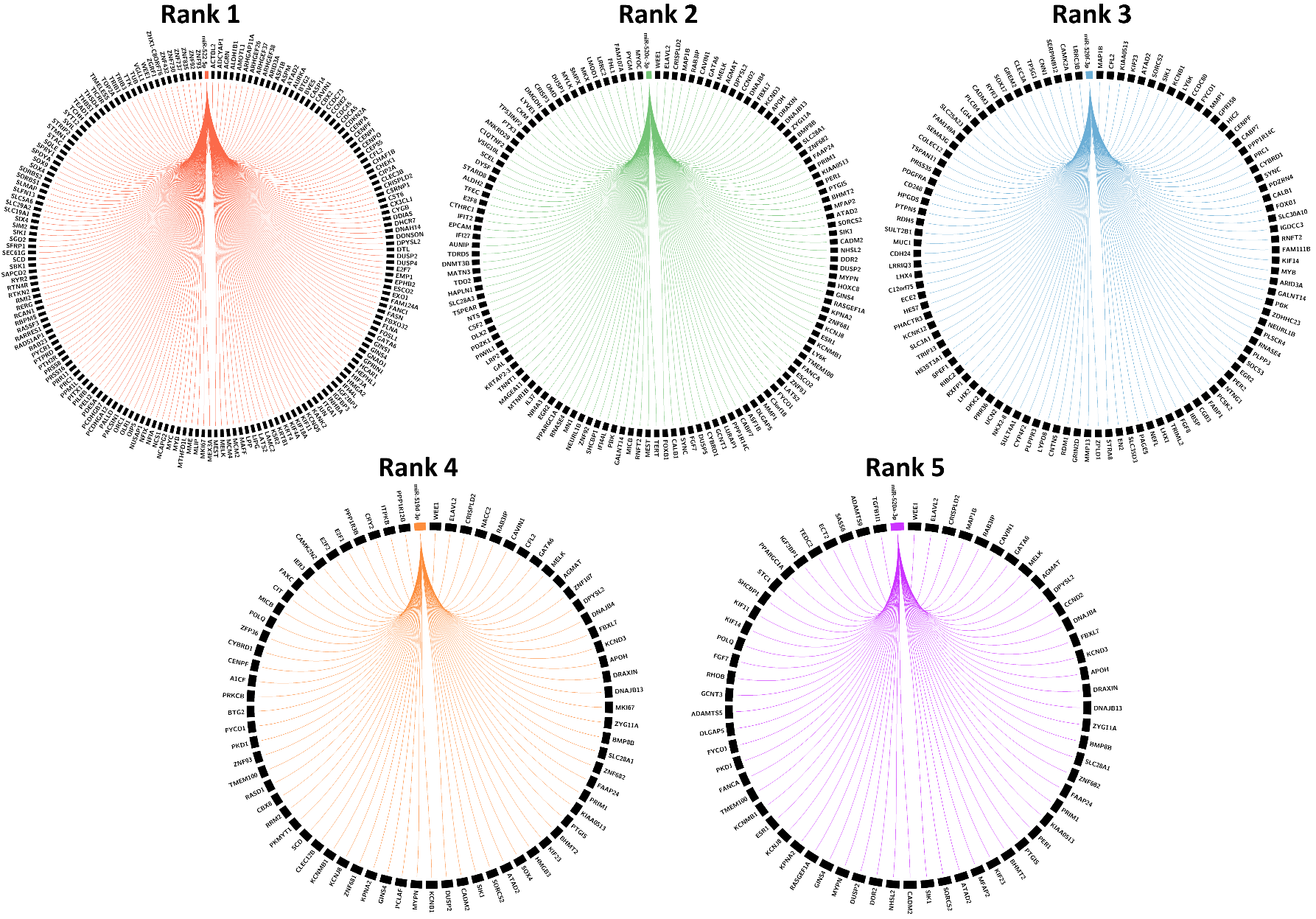


**Supplementary Fig. 5** Interaction network of C19MC members and their target genes. High confidence miRNA target genes were predicated with the help of multiMiR R/Bioconductor package. The top miRNAs were prioritised according to the highest number target. The miRNA-mRNA regulatory networks were visualized by using Circos tool.
